# Genome and Phylogenetic Analyses of *Trypanosoma evansi* Reveal Extensive Similarity to *T. brucei* and Multiple Independent Origins for Dyskinetoplasty

**DOI:** 10.1371/journal.pntd.0003404

**Published:** 2015-01-08

**Authors:** Jason Carnes, Atashi Anupama, Oliver Balmer, Andrew Jackson, Michael Lewis, Rob Brown, Igor Cestari, Marc Desquesnes, Claire Gendrin, Christiane Hertz-Fowler, Hideo Imamura, Alasdair Ivens, Luděk Kořený, De-Hua Lai, Annette MacLeod, Suzanne M. McDermott, Chris Merritt, Severine Monnerat, Wonjong Moon, Peter Myler, Isabelle Phan, Gowthaman Ramasamy, Dhileep Sivam, Zhao-Rong Lun, Julius Lukeš, Ken Stuart, Achim Schnaufer

**Affiliations:** 1 Seattle Biomedical Research Institute, Seattle, United States of America; 2 Swiss Tropical and Public Health Institute, Basel, Switzerland; 3 Department of Infection Biology, Institute of Infection and Global Health, University of Liverpool, Liverpool, United Kingdom; 4 Department of Pathogen Molecular Biology, Faculty of Infectious and Tropical Diseases, London School of Hygiene and Tropical Medicine, London, United Kingdom; 5 CIRAD, UMR-InterTryp, Montpellier, France; 6 Faculty of Veterinary Medicine, Kasetsart University, Bangkok, Thailand; 7 Centre for Genomic Research, Institute of Integrative Biology, University of Liverpool, Liverpool, United Kingdom; 8 Unit of Molecular Parasitology, Department of Biomedical Sciences, Institute of Tropical Medicine, Antwerp, Belgium; 9 Centre of Immunity, Infection and Evolution, University of Edinburgh, Edinburgh, United Kingdom; 10 Biology Centre, Institute of Parasitology, Czech Academy of Sciences, České Budějovice (Budweis), Czech Republic; 11 Faculty of Sciences, University of South Bohemia, Centre, České Budějovice (Budweis), Czech Republic; 12 Center for Parasitic Organisms, State Key Laboratory of Biocontrol, School of Life Sciences, and Key Laboratory of Tropical Disease Control of Ministry of Education, Zhongshan School of Medicine, Sun Yat-Sen University, Guangzhou, People′s Republic of China; 13 Wellcome Trust Centre for Molecular Parasitology, Institute of Biodiversity, Animal Health and Comparative Medicine, College of Medical, Veterinary and Life Sciences, University of Glasgow, Glasgow, United Kingdom; 14 Canadian Institute for Advanced Research, Toronto, Canada; 15 Department of Global Health, University of Washington, Seattle, United States of America; 16 Institute of Immunology & Infection Research, University of Edinburgh, Edinburgh, United Kingdom; Yale School of Public Health, United States of America

## Abstract

Two key biological features distinguish *Trypanosoma evansi* from the *T. brucei* group: independence from the tsetse fly as obligatory vector, and independence from the need for functional mitochondrial DNA (kinetoplast or kDNA). In an effort to better understand the molecular causes and consequences of these differences, we sequenced the genome of an akinetoplastic *T. evansi* strain from China and compared it to the *T. b. brucei* reference strain. The annotated *T. evansi* genome shows extensive similarity to the reference, with 94.9% of the predicted *T. b. brucei* coding sequences (CDS) having an ortholog in *T. evansi*, and 94.6% of the non-repetitive orthologs having a nucleotide identity of 95% or greater. Interestingly, several procyclin-associated genes (PAGs) were disrupted or not found in this *T. evansi* strain, suggesting a selective loss of function in the absence of the insect life-cycle stage. Surprisingly, orthologous sequences were found in *T. evansi* for all 978 nuclear CDS predicted to represent the mitochondrial proteome in *T. brucei*, although a small number of these may have lost functionality. Consistent with previous results, the F_1_F_O_-ATP synthase γ subunit was found to have an A281 deletion, which is involved in generation of a mitochondrial membrane potential in the absence of kDNA. Candidates for CDS that are absent from the reference genome were identified in supplementary *de novo* assemblies of *T. evansi* reads. Phylogenetic analyses show that the sequenced strain belongs to a dominant group of clonal *T. evansi* strains with worldwide distribution that also includes isolates classified as *T. equiperdum*. At least three other types of *T. evansi* or *T. equiperdum* have emerged independently. Overall, the elucidation of the *T. evansi* genome sequence reveals extensive similarity of *T. brucei* and supports the contention that *T. evansi* should be classified as a subspecies of *T. brucei*.

## Author Summary

The single-cell parasite *Trypanosoma evansi* is the disease-causing trypanosome with the widest geographical distribution. The disease, called surra, has significant economic impact primarily due to infections of cattle, horses, and camels. Morphologically the parasite is indistinguishable from bloodstream stage *T. brucei*, a parasite causing sleeping sickness in humans and the disease nagana in animals. *T. brucei*, however, is strictly bound to sub-Saharan Africa where its obligate vector, the tsetse fly, resides. The lack of a complete mitochondrial genome in *T. evansi* further distinguishes this parasite from *T. brucei*. Important questions regarding the biology of *T. evansi* include how it escaped from Africa, whether this has happened more than once, and how exactly it is related to *T. brucei*. To help answer these questions we have sequenced the *T. evansi* nuclear genome. Our phylogenetic analysis demonstrates that *T. evansi*, and the closely related horse parasite *T. equiperdum*, evolved more than once from *T. brucei*. We also demonstrate extensive similarity to *T. brucei*, including the maintenance of numerous genes that *T. evansi* no longer requires. Therefore, despite the significant functional and pathological differences between *T. evansi* and *T. brucei*, our analysis supports the notion that *T. evansi* is not an independent species.

## Introduction

Trypanosomatid parasites *Trypanosoma evansi* and *T. equiperdum* are responsible for animal diseases with extensive pathological and economic impact and closely related to the *T. brucei* group [1], [2]. The latter includes three subspecies: the human parasite *T. b. rhodesiense*, the zoonotic parasite *T. b. gambiense*, and the animal parasite *T. b. brucei*. Together *T. brucei*, *T. evansi*, and *T. equiperdum* comprise the subgenus *Trypanozoon*. The exact nature of the phylogenetic relationship between these three species has been the subject of ongoing debate, with some evidence suggesting that *T. evansi* and *T. equiperdum* are monophyletic and other evidence suggesting that they are polyphyletic and have emerged multiple times from *T. b. brucei* [3]–[6]. Trypanosomatids are a family within the protist group Kinetoplastida, the eponymous feature of which is a large and complex network of circular DNAs (kinetoplast or kDNA) inside their single mitochondrion. Two key biological features distinguish *T. evansi* and *T. equiperdum* from the *T. brucei* group. Firstly, their transmission is independent from the tsetse fly as obligatory vector. *T. evansi* is predominantly transmitted by biting flies and causes surra in a wide variety of mammalian species (the name of the disease varies with geographical area), while *T. equiperdum* causes a sexually transmitted disease called dourine in horses [1], [3], [7]. The altered mode of transmission has enabled both parasites to escape from the sub-Saharan tsetse belt and become the pathogenic trypanosomes with the widest geographical distribution. Secondly, all strains of *T. evansi* and *T. equiperdum* investigated so far are dyskinetoplastic, i.e., lacking all (akinetoplastic) or critical parts of their kDNA [8]. The loss of kDNA is thought to lock *T. evansi* and *T. equiperdum* in the bloodstream life cycle stage, presumably because the absence of kDNA-encoded components of the oxidative phosphorylation system prevents ATP generation in the tsetse midgut [9]. Nonetheless, whether dyskinetoplasty preceded the switch to tsetse-independent transmission or vice versa is unresolved [8], [10], [11].

The kDNA that comprises the mitochondrial genome in *T. brucei* consists of numerous concatenated circles of two kinds: maxicircles that encode genes primarily involved in oxidative phosphorylation and minicircles that encode guide RNAs (gRNAs) [12]. The majority of maxicircle mRNAs undergo RNA editing to insert or delete uridine residues as specified by template gRNAs in a process catalyzed by multiprotein complexes called editosomes [13]–[15]. One kDNA-encoded transcript that requires editing is the F_1_F_O_-ATPase subunit 6, which is essential in both bloodstream and insect stage *T. brucei* [16]–[19]. However, it has recently been shown that mutations found in the nuclear-encoded ATPase subunit γ of some *T. evansi* and *T. equiperdum* strains can compensate for the loss of kDNA, explaining their viability [20].

In an effort to better understand the causes and consequences of tsetse-independent transmission and kDNA-independent viability, we sequenced the genome of *T. evansi* strain STIB805. This strain was isolated in 1985 from an infected water buffalo in the Jiangsu province of China, shown to completely lack kDNA (i.e. to be akinetoplastic), and suggested to belong to a possibly clonal group of *T. evansi* with worldwide distribution [4], [21], which is why it was chosen for this study. The comparative genome analysis between this strain and the *T. b. brucei* TREU 927/4 strain reference genome [22] revealed extensive similarities. While the sizes of the chromosomes differ between *T. evansi* and *T. brucei*, the gene content within their respective genomes are largely similar, as 92.7% of *T. evansi* CDS have an identifiable ortholog in *T. brucei*. Analysis of *T. evansi* variant surface glycoprotein (VSG) sequences shows broad conservation of N-terminal sub-types, with extensive phylogenetic similarity and no evidence of any species-specific expansion of clades. An analysis of *T. evansi* CDS corresponding to the identified *T. brucei* mitochondrial proteome revealed that virtually all are retained, despite the lack of requirement in an akinetoplastic trypanosome for respiratory complexes I-IV or any proteins involved in maintenance or expression of the mitochondrial genome. Phylogenetic analyses with several genetic markers conclusively show that extant strains of *T. evansi* or *T. equiperdum* are not monophyletic and evolved on at least four independent occasions. Together, the results presented here show few critical differences between *T. evansi* and *T brucei*, indicating that dyskinetoplasty and concomitant tsetse-independent transmission are significant phenotypic changes underpinned by relatively subtle genomic alterations.

## Materials and Methods

### Ethics statement

The rearing of animals was regulated by Czech legislation (Act No 246/1992 Coll.). All housing, feeding and experimental procedures were conducted under protocol 90/2013 approved by Biology Centre, Czech Academy of Sciences and Central Commission for Animal Welfare of the Czech Republic.

### Whole genome sequencing

Trypanosomes (*T. evansi* strain STIB805) were purified from mice by DEAE (DE52) cellulose [23]. Total DNA was extracted as described elsewhere [24]. Briefly, the cells were lysed using SDS, and incubated with proteinase K and RNase. DNA was harvested after phenol extraction and ethanol precipitation. Four runs of single-end 454 sequencing plus 2 runs of paired-end 454 sequencing were obtained using GS FLX(+) System following the manufacturer's instruction (Roche) and generated 1,904,327 reads (225,826 paired end, 1,678,501 single end) [25]. Approximately 10 µg of genomic DNA was sheared by nebulization into desired fragments sizes (∼400 bp for single-end 454, ∼3 kb for paired-end) and adaptor oligos ligated to create the library for sequencing. Additional sequence data was obtained by shearing genomic DNA to ∼200–300 bp fragments sizes for sequencing on an Illumina GAIIx producing 19,701,740 tags with an ordered read length of 76mers. Illumina reads for *T. b. brucei* strains TREU 927/4 and Lister 427 (provided by the Wellcome Trust Sanger Institute, Hinxton, UK) were downloaded from the European Nucleotide Archive (accession nos. ERX009953 and ERX008998).

### Sequence assembly and SNP calling

Genome assemblies and identification of sequence polymorphisms (SNPs and indels) were carried out with CLC Genomics Workbench (CLC bio). Reads for *T. evansi* STIB805, *T. b. brucei* TREU 927/4 and *T. b. brucei* Lister 427 were mapped against the *T. brucei* TREU 927/4 version 4 reference (Tb927) using the following mapping parameters: global alignment, similarity fraction  = 0.9, length fraction  = 0.5, insertion cost  = 3, deletion cost  = 3, mismatch cost  = 2. *De novo* assemblies for each strain were created using either all reads or those binned during the reference-based assemblies, using the following parameters: automatic word size  =  yes, bubble size  = 50, similarity fraction  = 0.9, length fraction  = 0.5, deletion cost  = 3, insertion cost  = 3, mismatch cost  = 2. *De novo* contigs were aligned to Tb927 using standalone NCBI BLAST version 2.2.25 and Artemis Comparison Tool release 12.0 [26]. For RPKM (reads per kilo base per million) analysis, aligned STIB805 and TREU 927/4 reads were assigned to sequential 1-kb bins along the length of the Tb927 reference. For each chromosome, the log2 ratio of binned reads from the two read-sets was calculated for each bin, and normalized to a median log2 ratio of 0 by offsetting all values by the median log2 ratio. SNPs and indels were called with CLC bio using the following parameters: minimum coverage  = 10, maximum coverage  = 100, minimum variant frequency  = 30%, minimum central quality  = 20, minimum average quality  = 15.

### Gene prediction and annotation

Coding sequence prediction of *T. evansi* genome was done using a combination of *de novo* gene prediction approach and reference based gene transfers. The Rapid Annotation Transfer Tool (RATT) was used to transfer the gene boundaries and functional annotations from Tb927 onto *T. evansi* chromosomes (target hereafter) [27]. To prevent the transfer of paralogs to the same target region, thus resulting in multiple overlapping/duplicate gene calls, the annotation transfer was performed pairwise between one chromosome from Tb927 and the corresponding chromosome from the target. This resulted in the transfer of more than 96.4% (9427/9776) of genes from Tb927_v4 onto TevSTIB805ra. Though we observed extensive conservation of synteny between Tb927 and target genomes, this approach would likely have missed genes in targets that were shuffled by chromosomal fission and fusion that occurred during evolution. We performed another RATT transfer considering all Tb927 chromosomes and one target chromosome at a time. We then parsed out genes that were predicted uniquely by this approach (i.e. from regions where the initial RATT transfer failed to predict any genes). Combining these two approaches allowed us to predict genes that were shuffled across the chromosomes while avoiding predictions that overlap each other. We then used an in-house consensual *de novo* gene prediction suite called 'AutoMagi' to predict protein coding genes from *T. evansi* [28]. AutoMagi internally uses three gene prediction algorithms (genescan, testcode, codonusage) and predicts a consensus gene model out of individual gene predictions. The codon usage table required by AutoMagi was generated by ‘cusp’ using the RATT output of *T. evansi* STIB805 assembly [29]. We then used an in-house built prolog system to combine the *de novo* gene predictions and reference-based gene predictions. The genes that are unique to RATT predictions were automatically included in the final set. Genes that are unique to AutoMagi were compared with the NCBI non-redundant (NR) database (accessed 23 February 2011) using 'BLASTp' (default criteria: EXPECT = −10, WORD SIZE = −3, MATRIX_NAME =  BLOSUM62, GAP COST =  Existence:11 Extension:1) algorithm. All of the 'BLASTp' results were reviewed manually, and genes meeting the following two criteria were retained: (a) an E-value of 5e-6 or lower to the matched NR sequence; (b) coding strand identical to nearest neighbors on either side (i.e. on same poly-cistronic unit). This manual curation removed 165 putative CDS from making into the finalized TevSTIB8805ra.

The gene calls that overlapped between RATT and AutoMagi were subdivided into the following 4 groups. 1) Identical: overlapping exactly, 2) AM_subsetof_RATT: AutoMagi prediction is entirely contained within RATT prediction, 3) RATT_subsetof_AM: RATT prediction is entirely contained within AutoMagi prediction 4) StaggeredOverlap: both predictions overlap in a staggered fashion. In the first two cases (Identical & AM_subsetof_RATT), AutoMagi gene calls were ignored and RATT models were retained. In the third case (RATT_subsetof_AM) coordinates from AutoMagi were combined with annotation information from the RATT model. Genes in the fourth category (Staggered) were subjected to a thorough manual review process. The review process involved (i) 'BLASTp' search against NCBI's NR database to identify coordinates for queries and subject; (ii) a ClustalW multiple sequence alignment of both candidate genes with their potential homolog(s) from *T. brucei*. Manual review of BLAST and ClustalW was performed in each case to decide either to split/merge/choose one of the AutoMagi/RATT predictions. These newly derived coordinates were then combined with the annotation information from the RATT model. GeneIDs of the final set of protein coding genes were unified and ordered from left to right end of the chromosome.

### Genome publication

Entire genome and all the predicted genes are publicly available at TriTrypDB (http://tritrypdb.org/tritrypdb/). The fastq files containing the Illumina read data for *T. evansi* STIB805 are available at: http://www.ebi.ac.uk/ena/data/view/ERA000101.

### Pulse-field gel electrophoresis

Bloodstream parasites at 2×10^8^ cells/ml were purified using DEAE cellulose (DE52) chromatography, and subsequently used to prepare chromosome blocks as previous described [30]. DNA from *T. b. evansi* and *T. b. brucei* cells was embedded in low-melt agarose blocks (final concentration of 5×10^7^ cells/ml) according to [31], and was resolved using a 1% Megabase agarose (Bio-Rad) gel with 0.5X TBE buffer in the CHEF-DRIII system (Bio-Rad). *S. cerevisiae* DNA was used as a size marker. Pulse field gel electrophoresis (PFGE) was run at 14°C under the following conditions: switch time A increased from 28.6 s to 228 s for 24 hrs, followed by switch time B, with increase from 28.6 s to 1,000 s for another 24 hrs. The angle was set to 120° and voltage gradient to 3 V/cm. The PFGE gel was stained with ethidium bromide after the run.

### VSG sequence retrieval and alignment

VSG sequences were extracted from the genome assembly using hidden markov models (HMM) and HMMER 3.0 [32]. HMMs were constructed for a- and b-type VSG respectively using multiple sequence alignments of *T. brucei* TREU 927/4 and *T. b. gambiense* DAL972 sequences [33]. All open reading frames>100 bp were marked up and the predicted amino acid sequences were searched for matches to either HMM using HMMER 3.0. Significant matches were checked manually to ensure that each VSG was complete and gene boundaries were correct. Intact VSG were extracted and aligned approximately using Clustal X [34]. The aligned sequences were combined with existing alignments of *T. brucei* TREU 927/4 a- and b-type VSG [35] and modified by eye.

### VSG phylogenetic analysis

C-terminal domains were trimmed (due to recombination these present an inconsistent phylogenetic signal), resulting in a- and b-VSG alignments of 470 and 492 characters, respectively. Neighbour-joining trees were estimated for each amino acid sequence alignment using PHYLIP v3.6, with a JTT rate matrix and 100 non-parametric bootstrap replicates. A frequency distribution of species-specific clade sizes, (i.e. three *T. evansi* VSG clustered together with a *T. brucei* TREU 927/4 sequence as the sister lineage has a clade size of 3) was calculated to express the degree of intercalation of sequences from the two strains.

### VSG ortholog divergence

Putative VSG orthologs were extracted from the phylogenies. In situations where single VSG from *T. evansi* STIB805 and *T. brucei* TREU 927/4 were sister taxa, supported by a bootstrap value>95, these genes were interpreted as orthologs. The rates of synonymous and non-synonymous nucleotide substitutions per site were estimated for these orthologous pairs. The ratio of these rates (*ω*) was estimated using *Ka*/*Ks* Calculator v1.2 [36] using GY and MS methods. For comparison, this was repeated for 151 pairs of non-VSG orthologs chosen at random.

### Phylogenetic analyses

The dihydrolipoamide dehydrogenase (LipDH) CDS (Tb927.11.16730) was PCR-amplified from total parasite DNA using primers 5′-ATA AAG CTT ATG TTC CGT CGC TGC-3′ (forward) and 5′-ATA AGA TCT TTA GAA GTT GAT TGT TTT GG-3′ (reverse) and Phusion polymerase (New England Biolabs, NEB). In cases where direct sequencing of the amplicon revealed heterozygosity, sequence information for individual alleles was obtained after cloning. After removal of the primer sequences, LipDH CDSs were aligned using ClustalX [37]. Phylogenies were reconstructed using the program MrBayes [38] via the Topali platform [39], implementing the GTR substitution model and a discrete gamma rate distribution model with four rate categories (to account for rate heterogeneity among sites) as the most appropriate nucleotide substitution model. Four independent MCMC chains of 1×10^6^ generations were sampled every 100^th^ generation. A 50% majority rule consensus tree was derived after the first 25% of trees were discarded as burn-in. The ATP synthase γ subunit sequence (Tb927.10.180) was PCR-amplified from total parasite DNA with primers 5′-GCG GAA TTC GAA GCA GAT GAC ACC TAA-3′ (forward) and 5′-GCG GAA GAC CTT GCT GCG GAG CCA CTC T-3′ or 5′-GGC GAC ATT CAA CTT CAT-3′ (reverse) and Phusion polymerase (NEB). The sequence was determined by direct amplicon sequencing or, in cases of heterozygosity, after cloning. A partial 812-bp sequence of cytochrome oxidase 1 (COX1) was obtained by PCR and used for phylogenetic analysis as previously described [40]. Briefly, we assessed phylogenetic relationships among *T. equiperdum* and *T. brucei* isolates using a haplotype network constructed using the statistical parsimony approach implemented in TCS v. 1.21 [41]. Subnetworks were created with 95% confidence limit and then unconnected subnetworks>10 mutations apart were connected by relaxing the confidence limit. To verify that the haplotypes containing *T. equiperdum* isolates were on phylogenetically distinct branches, we estimated a phylogenetic tree using the Bayesian approach implemented in MrBayes v. 3.2 [42]. PartitionFinder v. 1.0.1 [43] determined the Hasegawa, Kishino and Yano nucleotide substitution [44] with invariant sites (HKY+I) without partitioning by codon positions as the most appropriate model for the MrBayes analysis. Microsatellite genotyping was carried out exactly as described previously [40]. Briefly, isolates were typed for eight microsatellite markers [45]. Principal component analysis (PCA) was performed in R using the package adegenet, as described [40].

## Results

### Pulse-field gel electrophoresis (PFGE)

**Figure 1:**
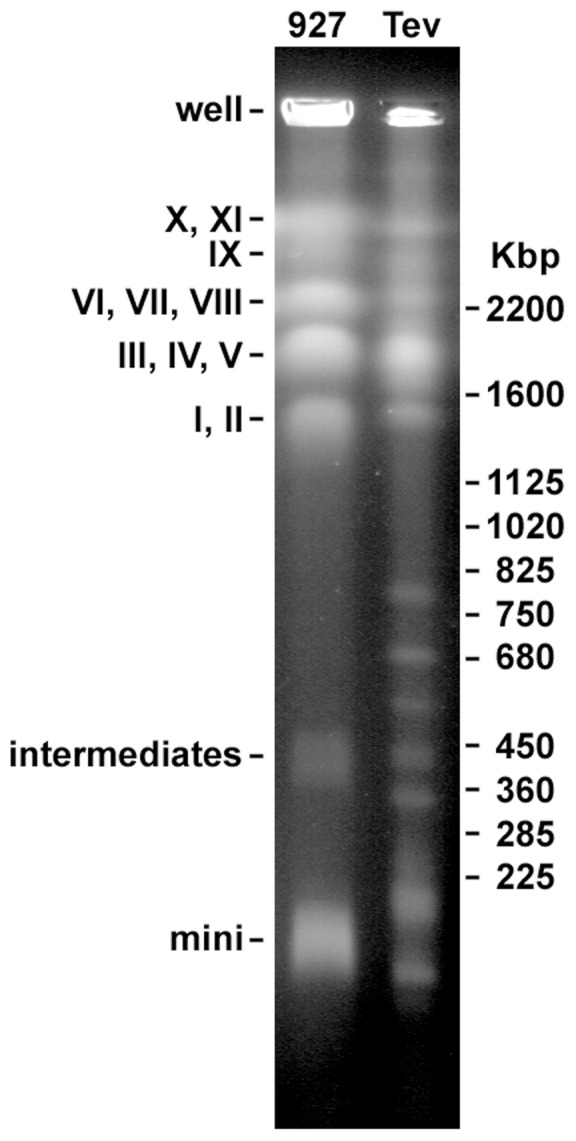
Pulse-field gel electrophoresis comparing chromosomes of *T. evansi* STIB805 (Tev) and *T. b. brucei* TREU 927/4 (927). While the sizes of megabase chromosomes are largely similar, differences in the intermediate and minichromosomes (825 kbp and smaller) are evident between *T. evansi* and *T. brucei*. Although *T. brucei* chromosomes I-XI were not unambiguously identified, labels to the left of the gel indicate bands consistent with the expected *T. brucei* chromosome sizes [46], as well as intermediate and minichromosomes. The signal at the top of the gel is from the well, as indicated.

PFGE was performed to visualize the pattern of chromosomes in the akinetoplastic *T. evansi* STIB805 strain. Multiple bands corresponding to megabase chromosomes ranging in size from ∼1 to ∼5 Mb could be visualized (Fig. 1). The most noticeable differences in the chromosomal pattern in comparison to *T. brucei* are the five intermediate chromosome bands that range from ∼300 kb to ∼800 kb. Additionally, two size groups of minichromosomes (∼100 kb and ∼200 kb) were observed in *T. evansi* STIB805. Such a degree of variability is within the range observed among strains of *T. b. brucei* [46].

### *T. evansi* reference-based genome

Genomic DNA isolated from *T. evansi* STIB805 was subjected to both 454 and Illumina sequencing, generating a combined total of 21,445,221 reads. Using the *T. brucei* TREU 927/4 genome sequence [22] (version 4; from here onwards abbreviated Tb927 for convenience) as a scaffold, a reference-based assembly of these *T. evansi* reads was generated. This assembly, called TevSTIB805ra, incorporates 17,856,165 reads and has an average coverage of 57.2 reads per nucleotide across the entire assembly and of 48.4 reads per nucleotide for the ‘core regions’ [i.e. regions excluding telomeres, subtelomeres, and internal regions that consist of repetitive coding sequences such as VSGs, expression site-associated genes (ESAGs) or retroposon hot spot genes (RHS)].

An assembly approach based on the genome of a closely related reference strain allows the reliable identification of homologous gene pairs, of any differences that might exist between these sequences, and of genes that are missing or highly diverged in the genome of interest. This approach has limited power in identifying structural differences between genomes and, by definition, cannot identify sequences that are present in the genome of interest but not in the reference genome. For that reason we have supplemented the reference-based approach with the analysis of contigs that were assemble *de novo*. As detailed below, this allowed the confirmation of Tb927 genes that are absent in in *T. evansi* STIB805 and the identification of candidate genes that might be present in the latter but absent in the former. However, genome rearrangements that are not associated with differences in gene content will have been missed and the *T. evansi* STIB805 genome as published on TriTrypDB may indicate gene synteny where it does not exist.

Annotation of the TevSTIB805ra *T. evansi* genome was performed using a combination of RATT and AutoMagi, followed by manual curation. RATT (Rapid Annotation Transfer Tool; [27]) identified likely orthologs in the *T. brucei* reference genome and transferred their functional annotations to the *T. evansi* genome, while AutoMagi predicted genes *de novo* using the consensus of three gene prediction algorithms (genescan, testcode, codonusage) [28]. A total of 10,110 CDS were identified, with 9368 CDS annotated as *T. brucei* orthologs by RATT and subsequent manual inspection (Table 1, columns C, E and F; S1 Table), and 742 CDS uniquely predicted by AutoMagi (Table 1, column D; S2 Table). Thus, 92.7% of the identified *T. evansi* CDS have an identified ortholog in *T. brucei*, while 7.3% were uniquely detected by *de novo* gene prediction. Analysis of the 742 AutoMagi CDS by BLAST searching of the GenBank non-redundant database revealed at least one hit (E-value ≤5e-6) for each of these CDS to a gene from a *Trypanosoma* species (*T. b. gambiense* 568 CDS; *T. b. brucei* 168 CDS; *T. equiperdum*, *T. vivax*, or *T. congolense* 6 CDS). The most common annotations among these BLAST hits were for ‘hypothetical unlikely’ (415 CDS) or other hypothetical (150 CDS) sequences (S2 Table). Orthologous sequences had not been annotated in the Tb927 reference, presumably due to more stringent criteria for gene calling. Thus, most if not all of the *de novo* predicted genes in TevSTIB805ra are also present in *T. brucei*.

**Table 1:** Chromosomal breakdown of CDS found in TevSTIB805ra compared to Tb927 reference.

	A	B	C	D	E	F	G	H
	CDS found in TevSTIB805ra	CDS found in Tb927	Orthologous CDS pairs (found in both TevSTIB805ra and Tb927)	CDS uniquely found in TevSTIB805ra by AutoMagi	CDS found in TevSTIB805ra chromosome with RATT homolog in a different Tb927 chromosome	Additional non-syntenic CDS in TevSTIB805ra with RATT homolog in the same Tb927 chromosome	CDS found in Tb927 but not found in TevSTIB805ra	CDS found in Tb927 chromosome with RATT homolog in a different TevSTIB805ra chromosome
**Chr01**	524	522	507	13	4	0	10	5
**Chr02**	364	326	318	28	18	0	8	0
**Chr03**	618	579	565	44	9	0	14	0
**Chr04**	602	573	558	36	8	0	15	0
**Chr05**	585	526	495	82	8	0	31	0
**Chr06**	590	574	543	40	7	0	31	0
**Chr07**	836	754	740	89	3	4	13	1
**Chr08**	878	828	807	64	6	1	20	1
**Chr09**	1299	1472	1208	82	5	4	258	6
**Chr10**	1743	1654	1624	114	5	0	29	1
**Chr11_01**	1907	1807	1762	127	14	4	42	3
**Chr11_02**	85	83	72	10	3	0	11	0
**Chr11_03**	79	78	57	13	9	0	21	0
***total***	10110	9776	9256	742	99	13	503	17

Column A shows all CDS found in TevSTIB805ra, and is the sum of Columns C, D, E, and F. Column B shows all CDS found in Tb927, and is the sum of Columns C, G, and H. Column C shows the number of orthologous CDS pairs shared by TevSTIB805ra and Tb927 without additional counts for duplicated CDS. Column D lists numbers of CDS predicted by AutoMagi for which no annotated CDS was found in the same location in the Tb927 reference. Column E shows the numbers of TevSTIB805ra CDS that had annotation transferred from a Tb927 CDS on a different chromosome. Column F shows the number of additional CDS found in TevSTIB805ra that arise from multiple tandem *T. evansi* CDS matching a single CDS within a similar tandem array in Tb927, instead of the syntenic CDS. Since a reference–based assembly by definition identifies the read that is the best match for the reference sequence, genes listed in columns E and F most likely correspond to sequences that are unannotated pseudogenes in Tb927, but are related to annotated genes in other locations. Column G shows Tb927 CDS for which no TevSTIB805ra CDS was identified. Column H shows the numbers of Tb927 CDS that had annotation uniquely transferred to a TevSTIB805ra CDS on a different chromosome, i.e. no annotation transfer of these Tb927 CDS was made to the same TevSTIB805ra chromosome. See Tables in Supporting Information for additional information.

**Figure 2:**
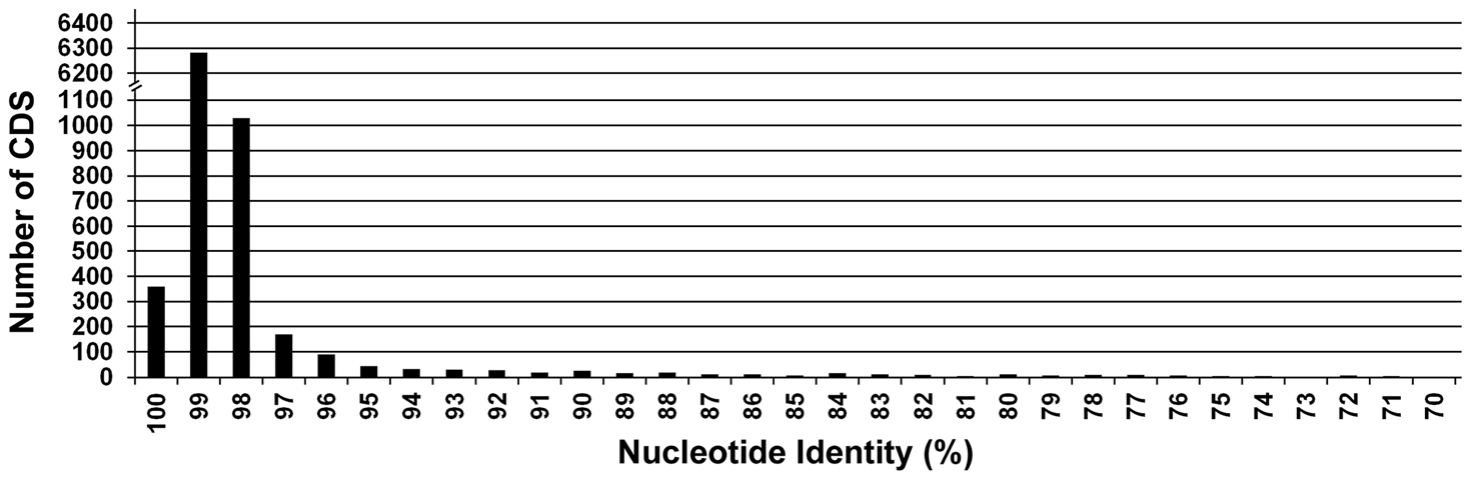
Frequency distribution of nucleotide identity between orthologous *T. evansi* vs. *T. brucei* coding sequences, including pseudogenes. This graph represents 8290 CDS with the highest identities out of 8421 total non-repetitive sequences analyzed.

Of the 9368 *T. evansi* CDS identified as *T. brucei* orthologs, 8421 CDS were classified as non-repetitive genes; the remaining 947 CDS were VSG, ESAG, RHS, or duplicate sequences. A comparison of the 8421 non-repetitive CDS between *T. brucei* and *T. evansi* revealed 7970 (94.6%) had a nucleotide identity of 95% or greater, 320 (3.8%) had a nucleotide identity between 70–95%, and 131 (1.6%) had a nucleotide identity less than 70% (Fig. 2; S1 Fig.). After RATT annotation transfer, a total of 503 (5.1%) Tb927 GeneIDs did not have identified orthologs in the TevSTIB805ra annotated genome (Table 1, column G; S2 Table). The majority of these (406) represent repetitive genes (e.g. VSG, ESAG, and RHS) or ‘hypothetical unlikely’ genes, which were not analyzed further. Five CDS correspond to predicted pseudogenes. *T. evansi* STIB805 orthologs for 49 Tb927 CDS were shown to be wholly or partially missing in TevSTIB805 reads that mapped to these gene loci (see below). *T. evansi* homologs for the remaining 43 Tb927 GeneIDs were ‘missed’ by RATT and AutoMagi, but were subsequently identified by manual examination of the *T. evansi* sequence. Thus, the majority of *T. brucei* CDS have extremely similar *T. evansi* orthologs, and very few *T. brucei* CDS were not found in *T. evansi*. This result is consistent with a very close phylogenetic relationship between these parasites.

The initial RATT approach only identified *T. evansi* CDS with an annotated Tb927 homolog in the syntenic position. To identify potential CDS in TevSTIB805ra that are syntenic to unannotated sequences in Tb927, and are homologous to annotated CDS in other (non-syntenic) locations, RATT was performed a second time by comparing each TevSTIB805ra chromosome to the entire Tb927 genome. This approach complemented *de novo* gene calling by AutoMAGI and identified 112 CDS in TevSTIB805ra (Table 1, columns E and F; S2 Table). The majority of these CDS were annotated as hypothetical proteins (64 CDS) or repetitive sequences such as VSG, ESAG, and RHS (39 CDS). For almost all loci, manual inspection revealed that either (i) a syntenic CDS existed in Tb927 that was unannotated or (ii) a syntenic, annotated CDS did exist in Tb927 but the presence of a highly similar sequence elsewhere caused a RATT artefact. An exception was TevSTIB805.6.770, where the syntenic sequence in Tb927 was found to be disrupted by frame-shifts, but in *T. b. gambiense* DAL972 to be annotated as Tbg972.6.420 (hypothetical protein, unlikely)

### Genes identified in *T. evansi* by de novo assembly

Two approaches were used to find genes in the *T. evansi* genome that may not be present in the *T. brucei* genome. Firstly, reads from *T. evansi* that did not match Tb927 were *de novo* assembled to make TevSTIB805dn and putative CDS called with AutoMagi (Table 2 and S2 Table). The 22 CDS with a homolog in Tb927 that was not a repetitive or hypothetical gene included multiple copies of pseudogenes for putative UDP-Gal/UDP-GlcNAc-dependent glycosyltransferase and lytic factor resistance-like protein, an adenosine transporter, calmodulin, a DNA topoisomerase, a leucine-rich repeat protein, ribulose-phosphate 3-epimerase, major surface protease B, and a galactokinase pseudogene. 36 of the 202 CDS that did not get a hit against Tb927 had a BLASTx hit in the NCBI non-redundant database: 23 CDS matched hypothetical *Trypanosoma spp.* and 9 appeared to be contaminants from other - mostly bacterial - organisms. The remaining 4 BLAST hits were for *T. vivax* RNA-dependent DNA polymerase and *T. evansi* VSGs (2 hits) and diminazene-resistance-associated protein.

**Table 2:** Genes identified in *T. evansi de novo* assemblies.

Assembly	Method	Number of CDS			Criteria
TevSTIB805dn^1^	AutoMAGI prediction	2165			total number
	BLASTp		1963		homolog in Tb927^2^
	BLASTp			1807	repetitive, e.g. RHS, VSG, ESAG
	BLASTp			134	hypothetical proteins
	BLASTp			22	others (see text)
	BLASTp		202		no homolog in Tb927^2^
	BLASTx			36/5^4^	hit in NCBI^3^
	BLASTx			166/17^4^	no hit in NCBI^3^
TevSTIB805dn_sub^5^	AutoMAGI prediction	1409			total number
	BLASTp		1292		homolog in Tb927^2^
	BLASTp			1208	repetitive, e.g. RHS, VSG, ESAG
	BLASTp			71	hypothetical proteins
	BLASTp			13	others (see text)
	BLASTp		117		no homolog in Tb927^2^
	BLASTx			18/1^4^	hit in NCBI^3^
	BLASTx			99/5^4^	no hit in NCBI^3^

1 3,589,056 reads from *T. evansi* STIB805 did not match the Tb927 reference were used for a *de novo* assembly. 3,056,932 reads could be assembled into 2590 contigs ≥500 bp with a combined length of 4,927,577 bp; (N50  = 978). Data are in S2 Table.

2 cut-off: BLASTp E-value ≤0.001.

3 cut-off: BLASTx E-value ≤0.001 in NCBI non-redundant protein database.

4 filtered for contigs with ≥5x minimum coverage.

5 1,951,783 reads from *T. evansi* STIB805 that matched neither the Tb927 reference, nor a *de novo* assembly of *T. brucei* TREU 927/4 Illumina reads, were used for a *de novo* assembly. 1,416,068 reads could be assembled into 2390 contigs ≥500 bp with a combined length of 3,239,882 bp (N50  = 1174).

Because Tb927 was created using a traditional sequencing approach and only covers the 11 large chromosomes [22], sequences found in the *de novo* assembly of *T. evansi* deep sequencing reads might not be unique to this strain or species, but rather reflect a difference in sequencing methodology or stem from intermediate-sized or mini-chromosomes. To address this, Illumina reads from sequencing *T. brucei* TREU 927/4 that also did not match Tb927 were *de novo* assembled into Tb927dn. Comparison of Tb927dn to TevSTIB805dn showed that 45.6% of the binned *T. evansi* reads matched to Tb927dn. The remaining *T. evansi* reads were then assembled into TevSTIB805dn_sub and putative CDS called with AutoMagi (Table 2 and S2 Table). Of the 84 BLASTp matches to non-repetitive genes, 71 are annotated as hypothetical proteins, 2 are lytic factor resistance-like proteins, 1 is a putative transporter, and 10 are annotated as putative (or pseudogene) UDP-Gal or UDP-GlcNAc-dependent glycosyltransferase. Glycosyltransferases in *T. brucei* are frequently found in subtelomeric regions that are difficult to assemble due to repetitive sequences, and are known to be highly variable among trypanosome strains [33], [47]. Of the 117 TevSTIB805dn_sub CDS for which no match was identified in Tb927, 18 had a BLASTx hit in the NCBI non-redundant database: 9 CDS matched hypothetical genes from various *Trypanosoma* species and 1 matched a diminazene resistance-associated protein that was previously suggested to convey diminazene aceturate (Berenil) resistance to certain strains of *T. evansi* [48]. Thus, gene prediction found very few CDS in *de novo T. evansi* assemblies that are not present in *T. brucei*, and analysis of these CDS revealed differences reminiscent of those observed among strains of the same species.

When *T. evansi* STIB805 *de novo* contigs were filtered for minimum coverage of at least 5x, the number of genes without obvious homologs in Tb927 was reduced considerably (Table 2). To test the potential absence of these genes in *T. b. brucei* more rigorously, short read data sets for strains TREU 927/4 and Lister 427 were searched for matches to the 22 candidates from TevSTIB805dn and the 6 candidates from TevSTIB805dn_sub. Only seven CDS candidates remained where the sequence was either entirely absent from both *T. b. brucei* datasets, or did not contain an undisrupted ORF (S2 Table, highlighted). Whether these candidates are indeed functional CDS, and whether they are generally absent from *T. brucei ssp.* and present in *T. evansi*, and therefore are of potentially diagnostic value, requires further investigation. A non-redundant list of the ORFs that did not have a BLASTx hit in NCBI (200 ORFs in total) is provided as S1 Data File.

### CDS identified in *T. brucei* that are absent in *T. evansi*

A total of 49 CDS found in *Tb927* were not identified in TevSTIB805ra. These loci were analyzed individually by (i) specifically searching for matching *T. evansi* STIB805 reads (S2 Table); (ii) comparing reads per kilobase per million (RPKM) coverage plots of these regions for *T. brucei* and *T. evansi*; (iii) aligning contigs of a full *T. evansi* STIB805 *de novo* assembly to the respective regions in Tb927. These analyses confirmed the absence or disruption of several of these CDS in *T. evansi* STIB805 (including the iron/ascorbate oxidoreductase loci, the Tb927.9.7950/Tb927.9.7960 repeat, the Tb927.4.3200-.3270 region, both Tb927.8.490 and Tb927.8.500, and the Tb927.8.7300-.7330 region), as illustrated in S2–S9 Fig., with the procyclin loci described in detail below. These cases have in common that the loci in question show considerable variation among *Trypanozoon* strains and CDS appear to be absent in *T. b. brucei* Lister 427 and/or *T. b. gambiense* DAL972 as well [33]. The differences observed in *T. evansi* STIB805 compared to Tb927 therefore are unlikely to be relevant for kDNA loss or tsetse-independent transmission.

### Procyclin loci

**Figure 3:**
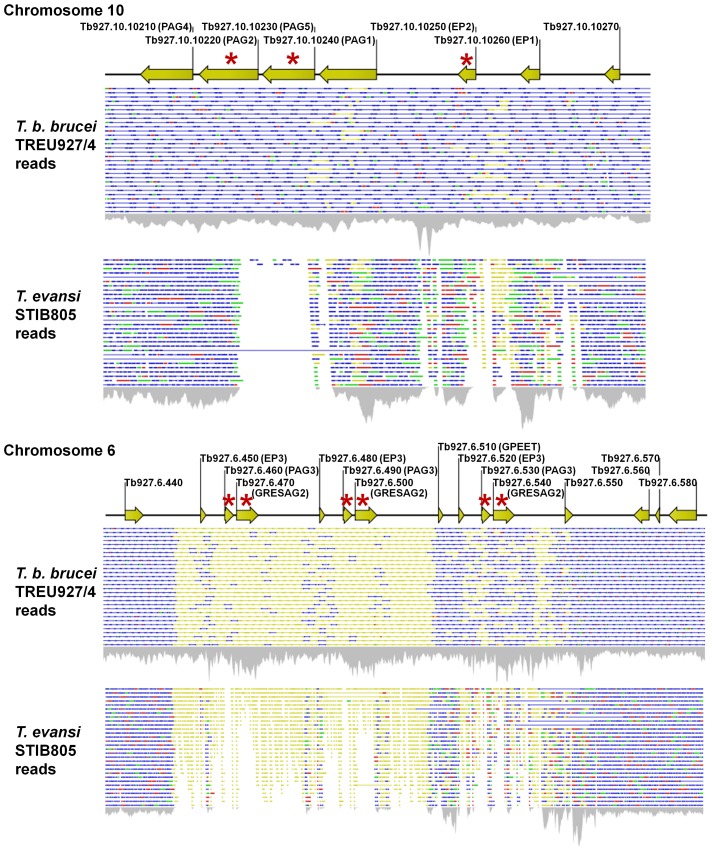
Schematic of reads from *T. b. brucei* TREU 927/4 and *T. evansi* STIB805 mapped on to the procyclin loci of *T. brucei*. CDS are denoted by yellow arrows, with Tb927 GeneIDs above and gene names in parentheses. Reads that could be uniquely mapped to the reference are colored blue (intact paired reads), green (single forward reads) or red (single reverse reads). Reads that could be mapped to more than one position in the Tb927 reference were placed randomly and are colored yellow. Red asterisks denote mutated or absent CDS in *T. evansi*. Both PAG5 and PAG2* are missing in *T. evansi*, and mutations are present in all copies of EP2, PAG3, and GRESAG2.

In *T. evansi* STIB805, the procyclin loci either lack or have disrupted versions of several CDS found in *T. brucei*. GPEET and EP procyclins and associated genes are encoded in loci on chromosomes 6 and 10, respectively, in various *T. brucei* strains [49]. Procyclin proteins are GPI-anchored coat glycoproteins that are expressed exclusively in the procyclic insect form of *T. brucei*, and they have been hypothesized to be involved in protection against tsetse fly midgut hydrolases [50]. Experiments in *T. brucei* have shown that knocking out all of the procyclin genes (Null mutants of GPEET and EP3 on chromosome 6; EP1 and EP2 on chromosome 10) causes no growth defect *in vitro* and permits completion of the entire life cycle, but causes a selective disadvantage during co-infection with wild type cells in the tsetse fly midgut [51]. These procyclin loci also contain procyclin-associated genes and a gene related to expression site associated gene 2 (PAG3 and GRESAG2 on chromosome 6; PAG1, 2, 2*, 4, and 5 on chromosome 10) [49]. The functions of the PAG proteins and GRESAG2 are unknown; although transcripts of PAG1–3 have been shown to increase during differentiation to procyclic forms, published experiments using cell lines with all PAG genes knocked out reported no obvious abnormal phenotypes *in vitro* or *in vivo* [49]. In multiple *T. brucei* strains, the chromosome 10 procyclin loci are heterozygous, with one chromosome containing EP1/EP2/PAG1/PAG5/PAG2*/PAG4 and the other chromosome containing EP1/EP2/PAG2/PAG4 (PAG2 being a fusion of the 5′ part of PAG1 and the 3′ part of PAG2*) [49], [52]–[56]. In *T. evansi* STIB805, chromosome 10 appears to be homozygous, with only the EP1/EP2/PAG2/PAG4 locus present, and the associated absence of the segment containing the 3′ part of PAG1, PAG5 and the 5′ part of PAG2* (Fig. 3). The full STIB805 *de novo* assembly contained a single 14.9 kb contig corresponding to the EP1/EP2/PAG2/PAG4 locus (S2 Fig.). Also on chromosome 10, the EP2 in *T. evansi* contains a stretch of 12 divergent amino acids in the domain N-terminal to the EP repeat; this region is highly conserved in *T. brucei* [57], [58]. Although the function of this domain is unknown, these 12 amino acids are found in all sequenced *T. brucei* genomes with very few variations. The chromosome 6 procyclin locus contains a triplication of three genes (EP3/PAG3/GRESAG2) in *T. brucei* TREU 927/4, with GPEET present only in front of the last unit (Fig. 3). Copy numbers of these genes may vary among *T. brucei* strains, as Southern analysis showed that these are single copy genes in the AnTat1.1 strain [49]. In TevSTIB805ra, coverage of this locus is much reduced (Fig. 3, S3 Fig.), indicating either divergence, reduced copy number, or both. The locus did not assemble into a single contig in the *de novo* approach and because of the repeat nature of this locus in Tb927, assigning sequence differences to particular gene copies was not possible. Nonetheless, several non-synonymous mutations in EP3 are evident, and the PAG3 and GRESAG2 genes have frameshifts and deletions. In contrast, analysis of a subset of other *T. evansi* orthologs to CDS shown to be upregulated in PF relative to BF *T. brucei* (Tb927.1.2310, Tb927.1.2350, Tb927.1.2560, Tb927.1.580, Tb927.10.10260, Tb927.10.10950, Tb927.10.4570, Tb927.11.16130, Tb927.11.8200, Tb927.4.1800, Tb927.4.1860, Tb927.4.4730, Tb927.5.1710, Tb927.5.2260, Tb927.6.510, Tb927.8.5260, Tb927.4.4730, Tb927.8.8300, Tb927.9.15110, and Tb927.9.8420) [59] detected no notable changes.

### Analysis of *T. brucei* mitoproteome orthologs in *T. evansi*

Any Tb927 gene encoding a product that is exclusive involved in (i) maintenance or expression of kDNA, (ii) life cycle progression, or (iii) insect-stage specific energy metabolism, e.g. oxidative phosphorylation, is redundant in *T. evansi*. Although many of these genes encode mitochondrial proteins, numerous other mitochondrial activities are expected to remain essential even in an akinetoplastic bloodstream trypanosome (see Discussion). Extensive proteomic analyses have identified 978 mitochondrial proteins in *T. brucei* [60]–[63]. A search of TevSTIB805ra and TevSTIB805dn identified orthologous gene sequences for all of these proteins (S6 Table). A detailed examination of a subset of genes representing categories i-iii above (with the exception of the F_1_ subunits of respiratory complex V, which remain essential in *T. evansi* [19]) suggests that the vast majority, if not all of these genes remain functional (see S6 Table for details). To obtain further evidence for or against mutational decay in these genes, we calculated *Ka*, *Ks*, and *Ka*/*Ks* for the following orthologous gene pairs in Tb927 and *T. evansi*: 208 CDS associated with kDNA expression or function; 942 unambiguous orthologous gene pairs for mitochondrial proteins; and 6331 unambiguous orthologous gene pairs corresponding to non-VSG and non-mitoproteome annotations (Table 3). These orthologous gene pairs were selected using greater stringency (reciprocal best matches in BLASTp searches with a minimum e-value threshold of 1×10^−4^) than the analysis shown in Fig. 2, to ensure that paralogous gene pairs were excluded (these would possibly increase *Ka* and *Ks* estimates and skew the distribution; S6 Table). We found no evidence of relaxed selection for *T. evansi* CDS associated with kDNA expression or function or the mitoproteome as a whole. Both *Ka* and *Ks* were lower for CDS associated with mitochondrial expression, and *Ka*/*Ks* was also lower relative to the genomic background, indicating that purifying selection is generally stronger among mitoproteome CDS, perhaps suggesting a higher proportion of essential genes compared to the control set. Furthermore, we made the same comparison between Tb927 and *T. b. gambiense* DAL972 (both of which retain functional kDNA) and the values for *Ka*, *Ks* and *Ka*/*Ks* show no significant differences (by t-test) with the values for *T. evansi*.

**Table 3:** Mean average *Ka*/*Ks* estimates for orthologous CDS pairs between Tb927 and either *T. evansi* STIB805 (*Tev*) or *T. b. gambiense* DAL972 (*Tbg*).

	n	*Ka*		p	*Ks*		p	*Ka*/*Ks*		p
		*Tbg*	*Tev*		*Tbg*	*Tev*		*Tbg*	*Tev*	
**non-mitochondrial, non-VSG**	6331	0.0068	0.0066	0.6106	0.0169	0.0172	0.7099	0.3853	0.4554	0.3582
**kDNA expression or function**	208	0.0033	0.0032	0.9413	0.0135	0.0137	0.9007	0.2291	0.2264	0.9180
**mitoproteome**	942	0.0037	0.0036	0.5203	0.0145	0.0141	0.4381	0.2602	0.2629	0.8586

Number of CDS pairs analyzed (n) for each category represents a subset of CDS that are unambiguous orthologs with reciprocal best match by BLASTp. Analysis by t-test of the values for *Ka*, *Ks*, and *Ka*/*Ks* for the *T. evansi* and *T. b. gambiense* isolates relative to Tb927 show no significant differences (p).

### VSG repertoire

**Figure 4:**
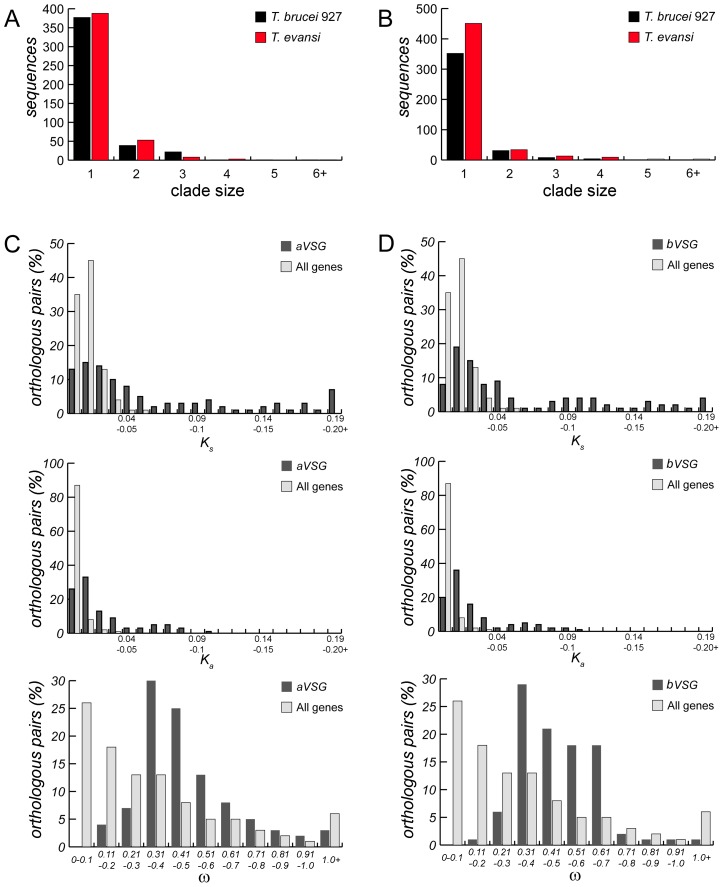
Diversity of a-type and b-type VSG between *T. b. brucei* TREU 927/4 and *T. evansi* STIB805 compared. Histograms showing a-type VSG (A.) or b-type VSG (B.) distributions of strain-specific clade size in *T. b. brucei* (black bars) and *T. evansi* (red bars) as defined by the phylogeny (see S10 Fig.). Frequency distributions of a-type VSG (C.) or b-type VSG (D.) synonymous (*Ks*) and non-synonymous (*Ka*) substitution rates per site, and the ω (*Ka/Ks*) for orthologous pairs of *VSG* (a-type n = 151; b-type n = 112), as defined by the phylogeny, in relation to values for unambiguous non-VSG orthologous pairs (n = 6331).

The predicted protein structures of VSGs in *T. evansi* STIB805 conform to the canonical structures in *T. b. brucei* and *T. b. gambiense*, and include all five recognized N-terminal sub-types (N1-5). In *T. evansi* STIB805 we identified 525 a-type VSGs (i.e. N-1-3 and 5; S2 Data File) and 505 b-type VSGs (i.e. N4; S3 Data File); of these 453 (86%) and 451 (89%), respectively, are full-length. Given that the assembly of subtelomeric and mini-chromosomal regions is fragmentary, these numbers may underestimate the real number of VSGs, although the total is comparable with both *T. b. brucei* TREU 927/4 [22] and *T. b. gambiense* DAL972 sequences [33]. The VSG repertoire is largely conserved between the *T. b. brucei* TREU 927/4 reference and *T. evansi* STIB805, as the VSGs are interspersed among each other in neighbour-joining molecular cladograms (S10 Fig.). Another indication of the similarity of the VSG repertoire is that very few clades of strain-specific VSG are larger than 2–3 (Fig. 4a and b). Large clades of VSG from a single genome would suggest divergence of VSG repertoire through gene duplication. Hence, 376 and 384 ( = 85.1%) *T. evansi* STIB805 and *T. b. brucei* TREU 927/4 a-VSGs, respectively, are most closely related to an ortholog in the other strain, or are paraphyletic to a clade of such sequences (i.e. strain-specific clade size  = 1). Only 40 *T. evansi* STIB805 VSGs and 51 *T. b. brucei* TREU 927/4 VSGs form a clade with a paralog from the same genome (i.e. strain-specific clade size  = 2), suggesting a single gene duplication since the strains separated. The same patterns occur with b-VSGs. Orthology between VSGs does not mean that they are unaffected by recombination, only that enough sequence homology persists for two orthologs to cluster together. Indeed, among 151 putative a-VSG orthologs 33 (21.8%) have dissimilar C-terminal types, while among 112 b-VSG orthologs 32 (28.6%) showed similar evidence for recombination having occurred since these two strains split from their common ancestor. Thus, analysis of the VSG repertoire reveals no evidence for the evolution of specific VSG gene clusters or subfamilies in *T. evansi*, similar to what had been observed for *T. b. gambiense* [33].

### VSG sequence evolution

This comparison of orthologous VSGs from *T. evansi* STIB805 and *T. b. brucei* TREU 927/4 provides an overview of the molecular evolutionary forces incident on the VSG archive. Fig. 4c and d show that substitution rates among orthologous VSGs are typically higher than the background defined by comparison to the 6331 non-VSG orthologous gene pairs previously used in the mitoproteome analysis above. The mean average rate for VSG is significantly greater than other genes (*p*<0.05) and comparison of *Ka*/*Ks* (*ω*) explains this. The distribution of ω is left-skewed for non-VSG genes, which reflects the strong purifying selection on non-synonymous substitutions that typifies most genes that perform essential functions. The distribution for VSG is normal, indicating that purifying selection is much weaker on average, although still in effect. This difference in the distribution of *ω* is significant (*p*<0.005) and shows that VSGs evolve in a more neutral fashion than other genes, resulting in higher substitution rates. However, there is no more evidence for positive selection among VSGs than for 'normal' genes, which reflects our current understanding that VSG sequence evolution in *T. brucei* is driven predominantly by the diversifying effects of recombination [35], rather than directional selection driven by host immune responses.

### RoTat 1.2

Consistent with earlier reports [64] that have suggested RoTat 1.2 (NCBI accession AF317914) as a predominant and diagnostic *T. evansi* VSG, we identified a closely related ORF in STIB805. The first 1250 bp of a 1431-bp ORF on a 3.9-kb *de novo* contig are nearly identical to the published RoTat1.2 sequence, while the 200 bp at the 3′ end are nearly identical to Tb927.8.240, annotated as ‘VSG, degenerate’ in TriTrypDB (S11 Fig.). Thus, the gene in STIB805 had most likely obtained a different C-terminal region through recombination, an important mechanism in VSG evolution [35]. We did not identify any close homologs to the 5′ region of RoTat1.2 in the publicly available *T. b. brucei* TREU 927/4, *T. b. brucei* Lister 427 or *T. b. gambiense* DAL972 datasets.

### Single nucleotide polymorphisms (SNPs) between *T. evansi* and *T. brucei*

A comparison of the TevSTIB805ra to the Tb927 reference genome revealed 354,809 single nucleotide polymorphisms, 68,938 of which were non-synonymous in identified CDS. 32,892 of these non-synonymous SNPs were found in 904 CDS classified as repetitive (e.g. RHS, VSG, ESAG), while 36,046 were found in 6630 CDS from non-repetitive genes (S3 Table). This number is slightly lower than the ∼45,000 non-synonymous SNPs reported for *T. b. gambiense* orthologs of non-repetitive *T. b. brucei* TREU 927/4 genes [33]. Additionally, there were 45,269 short indels (insertions/deletions of up to eight nucleotides), of which 5544 were in CDS regions. Of these, 4963 (89.5%) were not an indel of mod 3, and would therefore be expected to cause a frameshift. 3247 (58.6%) of the indels were found in 656 CDS classified as repetitive, while 2297 (41.4%) were found in 1091 CDS from non-repetitive genes (S4 Table). The latter consisted of 1154 insertions, 1000 deletions, and 143 that were complex (a combination of insertion, deletion and/or SNP). The Tb927 reference contains information for one allele only for any heterozygous locus. We therefore also identified SNPs and indels for TREU 927/4 using publicly available Illumina reads and the same criteria that were applied for STIB805. We identified 26,522 non-synonymous SNPs and 1630 indels. Of these, 814 and 58, respectively, were homozygous, thus reflecting discrepancies between the original Sanger sequencing data and the Illumina reads. The cause of these discrepancies, which could arise from a number of potential sources, including DNA isolation from different cultures of the same strain, is unknown. Only 1267 non-synonymous allele variations (SNP or indel) in CDSs (repetitive or non-repetitive) were shared by STIB805 and TREU 927/4, 8710 variations affected the same position, but were different, and 89,043 affected a different position. All non-synonymous SNPs and indels identified in STIB805 and TREU 927/4 are compared in S5 Table, listed by chromosome and gene. In summary, while numerous small sequence variations exist between *T. evansi* STIB805 and the *T. b. brucei* reference genome, these are comparable in number to differences between the *T. b. brucei* and *T. b. gambiense* subspecies.

### Phylogenetic analysis with dihydrolipoamide dehydrogenase (LipDH) and ATP synthase subunit γ as genetic markers

The phylogenetic relationship of various *T. evansi* and *T. equiperdum* isolates to each other and to isolates of *T. brucei* subspecies is controversial. We compared *T. evansi* STIB805 CDSs with their *T. b. brucei* TREU 927/4 orthologs as retrieved from Tb927 in order to identify a genetic marker that would likely be informative for the elucidation of the phylogenetic origins of *T. evansi* and *T. equiperdum*, and for determining their evolutionary relationships with established *T. brucei* sub-species. Selection criteria included (i) sufficient SNPs over a length of 1–3 kb (to minimize number of internal primers necessary for amplification and sequencing), (ii) lack of paralogs, and (iii) minimal alignment gaps. One promising candidate, the LipDH gene (Tb927.11.16730), a shared component of four mitochondrial multi-enzyme complexes [65], was selected for sequence analysis. Both LipDH CDS alleles were amplified and sequenced from a total of 40 isolates from various geographical locations (13 *T. b. brucei*, 3 *T. b. gambiense* group 1, 4 *T. b. rhodesiense*, 5 *T. equiperdum*, 15 *T. evansi*; S7 Table). Since mutations in subunit γ of the mitochondrial ATP synthase complex were recently identified as important factors in the viability of *T. evansi* and *T. equiperdum*, the sequence for both γ alleles was also determined for all isolates.

**Figure 5:**
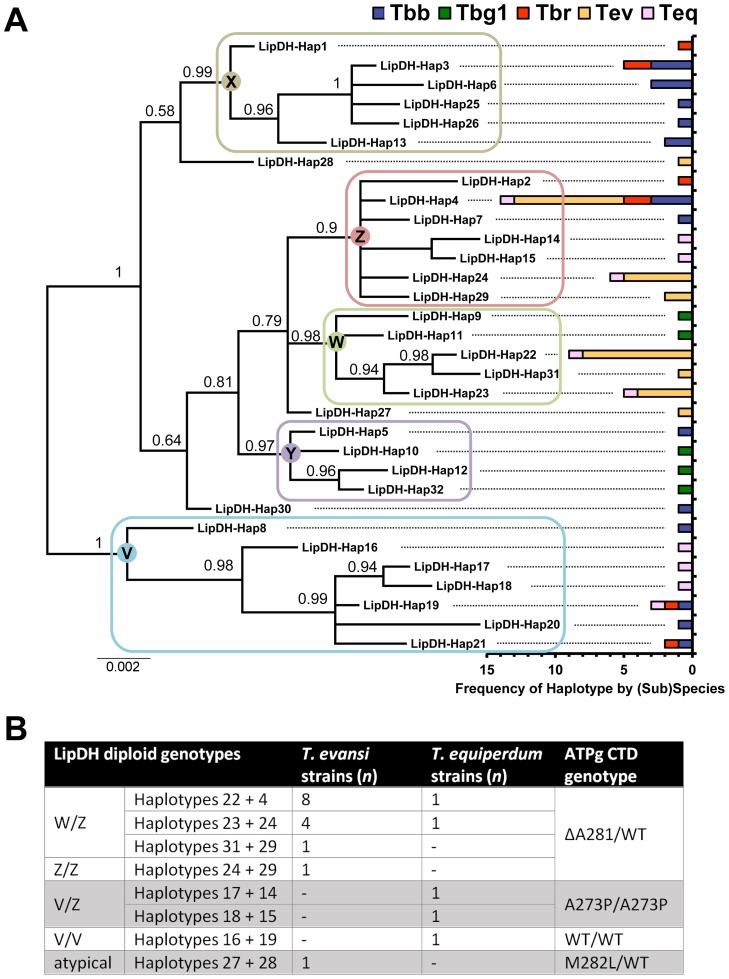
Bayesian phylogeny of *Trypanozoon* isolates based on the dihydrolipoamide dehydrogenase gene (LipDH; Tb927.11.16730). Panel A shows a mid-point rooted tree based on an alignment of 32 unique LipDH haplotypes, assembled from sequences derived from 13 *T. b. brucei* (Tbb), 3 *T. b. gambiense* type 1 (Tbg1), 4 *T. b. rhodesiense* (Tbr), 15 *T. evansi* (Tev) and 5 *T. equiperdum* (Teq) samples. Scale units for the phylogeny are substitutions per site. The chart illustrates the distribution of each haplotype among samples from each *Trypanozoon* taxon. Phylogenetic analysis grouped all but three of the haplotype sequences into one of five major clusters with strong support (posterior probabilities ≥0.9), which are referred to as clades V, W, X, Y and Z. Eight unique Tev/Teq genotypes were found, as summarized in panel B. Discounting minor sequence differences (1–2 SNPs) these were reduced to four major genotypes based on the position of haplotypes in the tree, which mirrored the type of mutation (A281Δ, M282L, A273P, WT) in the C-termini of the ATP synthase subunit γ in these the samples.

32 unique LipDH haplotypes were identified. The majority of strains (36/41) had two different alleles and four strains appeared to be homozygous at this locus, all of which were *T. b. brucei* (although it cannot be ruled out that the primers used were selective for one allele in these cases). Phylogenetic analysis (Fig. 5) showed all but three of the haplotype sequences fell into one of five major clusters with strong support (posterior probabilities ≥0.9), which we refer to as clades V, W, X, Y and Z. Importantly, 14 different haplotypes are derived from the *T. evansi*/*T. equiperdum* isolates, which are found in three different clades as well as outside of the clades. This feature is incompatible with monophyly of *T. evansi* or *T. equiperdum*. Some subspecies did have relatively restricted phylogenetic diversity, for example *T. b. gambiense* type 1 had only five closely related haplotypes and certain clades contained sequences from a restricted number of the subspecies; e.g. clade X only contained *T. b. rhodesiense* and *T. b. brucei*; clade Y included only *T. b. gambiense* type 1 and *T. b. brucei*. This may reflect relatively more recent origins of certain populations, bottleneck events and/or sampling bias, and is in line with most previous work ([40] and references therein).

Eight unique *T. evansi*/*T. equiperdum* genotypes (haplotype pairs) were found which, by disregarding minor sequence differences, were reduced to four major genotypes: W/Z, V/V, V/Z, or atypical (Fig. 5; S7 Table). These LipDH genotypes correlated very well with the four ATP synthase γ genotypes found in these strains (Fig. 5B; see also S7 Table).

The largest group (Group 1), consisting of 13 *T. evansi* isolates (including STIB805) and 2 *T. equiperdum* isolates, is characterized by LipDH genotype W/Z and γ genotype A281Δ/WT. The single exception, Tev48, had two Z haplotypes (Hap24+Hap29) distinguished by two SNPs, which may be a result of loss of heterozygosity (LOH) followed by two mutations, or it may represent a cloning artefact. Non-*T. evansi*/*T. equiperdum* strains that had sequences within these clades originated from both East and West Africa in the case of clade Z, and the *T. b. gambiense* samples from Ivory Coast in the case of clade W. The remaining *T. evansi*/*T. equiperdum* strains were split into three further groups according to the LipDH/ATP synthase γ genotypes as follows. Group 2 is characterized by LipDH V/Z and γ genotype A273P/A273P. The two *T. equiperdum* strains carrying this genotype (Teq21 and Teq22) were heterozygous for two alleles derived from well-separated clades: one ‘V’ allele (Hap17 or Hap18, 1 SNP difference) and one ‘Z’ allele (Hap14 or Hap15, 1 SNP difference). The divergence between the V and Z alleles (11 or 12 discriminating SNPs), suggests that these strains have evolved out of a *T. b. brucei* background that had relatively old and distinct LipDH alleles, possibly via a recombination event. Interestingly, the most closely related non-*T. evansi*/*T. equiperdum* alleles are found in current East African populations as well as in three *T. b. brucei* from Ivory Coast. Group 3 is characterized by LipDH V/V and γ genotype WT/WT. The one *T. equiperdum* strain with a WT γ genotype (Teq23 =  STIB841; probably synonymous with the OVI strain [5]) was heterozygous with two similar ‘V’ clade alleles that are closely related to the V alleles in Teq21, Teq22, Tbr02 (Kenya) and Tbb08 (Zambia) (S7 Table). Group 4 is characterized by atypical LipDH and γ genotype M282L/WT. This genotype was found in a single *T. evansi* strain (Tev42  =  KETRI 2479) with Hap27+Hap28 and could not be reliably grouped with any other strains.

An interesting aspect of the *T. evansi*/*T. equiperdum* genotypes is that most strains possess two divergent alleles that are more similar to those found in other, non-Tev/Teq strains than they are to each other. These non-Tev/Teq strains are themselves heterozygous, with second alleles that are clearly distinct from either of the Tev/Teq alleles. A clear example is the most common Teq/Tev LipDH genotype, W/Z, that is carried by 13 out of 15 *T. evansi* analyzed and 2 out of 5 *T. equiperdum* analyzed. This finding is incompatible with allelic divergence due to long-term clonal evolution, rather it shows that recombination between ancestral parasites occurred either at or immediately prior to the origin of this genotype which has subsequently undergone clonal expansion in the absence of the possibility of sexual exchange in the tsetse vector. Three of the four *T. b. rhodesiense* strains (Tbr01, 03, 04) also exhibit alleles split across different clades as does Tbb06. Interestingly, all Tbb except Tbb06 have alleles from the same clade, possibly indicative of inbreeding.

### Cytochrome oxidase 1 (COX1)

**Figure 6:**
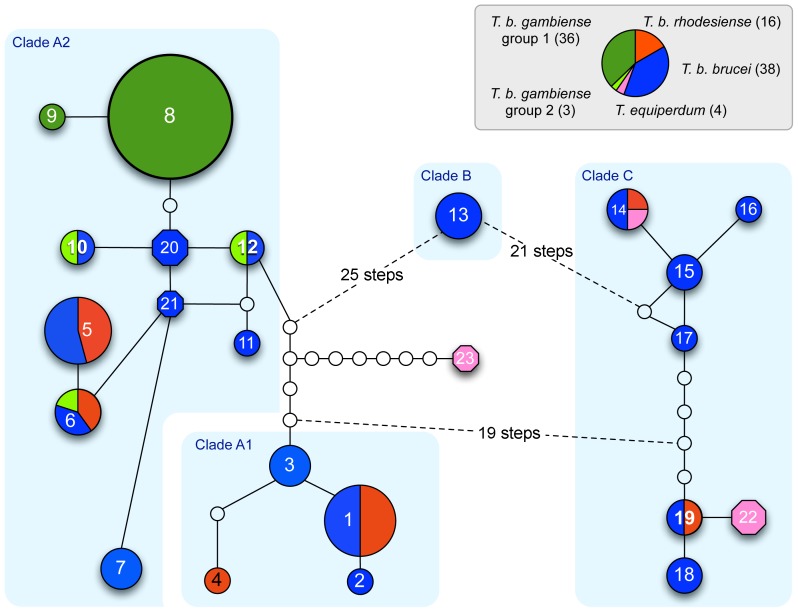
Maximum parsimony haplotype network showing phylogenetic relationships among *Trypanozoon* haplotypes using cytochrome oxidase 1 (COX1). Relationships among lineages of *T. b. brucei (blue)*, *T. b. rhodesiense (red)*, *T. b. gambiense* group 1 (dark green) and *T. b. gambiense* group 2 (light green) are shown in circles as in reference [40], with haplotypes new to this study shown as octagons, including two new haplotypes for *T. equiperdum* isolates (pink). The size of each circle or octagon is proportional to the frequency with which a particular haplotype was identified. Numbers in the circles and octagons correspond to haplotype ID. Empty circles indicate haplotypes that are inferred to exist but were not found. The light blue boxes correspond to the *T. brucei* clades defined in [40]. Haplotypes for samples new to this study are as follows (from left to right): 8 =  Tbg14; 5 =  Tbr01/Tbr04; 20 =  Tbb49/Tbb50; 21 =  Tbb51; 3 =  Tbb20; 2 =  Tbb38; 23 =  Teq24; 14 =  Tbr02/Teq23; 22 =  Teq21/Teq22.

The COX1 gene, encoded in the mitochondrial maxicircle DNA, was recently used as a highly informative marker to investigate the phylogeography of *T. brucei* subspecies [40]. Although most, if not all, *T. evansi* isolates lack a maxicircle, the presence of at least one *T. equiperdum* isolate with at least a partial maxicircle in three of the above groups prompted us to determine the COX1 haplotypes for these strains and to investigate their relationship with the *Trypanozoon* isolates from the earlier study (S7 Table). A maximum parsimony network analysis strongly supported the notion of three independent evolutionary origins for these three groups (Fig. 6). *T. equiperdum* STIB818 (Teq24; COX1 haplotype 23) links Group 1 with COX1 clade A, which is composed of isolates of all three *T. brucei* subspecies found across all of sub-Saharan Africa [40]. Although Groups 2 and 3, which share a related ‘V’ LipDH genotype, are both linked to COX1 clade C (composed of *T. b. brucei* and *T. b. rhodesiense* isolates from eastern and southern Africa), they are well separated within this clade, indicating independent evolutionary origins. *T. equiperdum* STIB841 (Teq23) shares both its COX1 haplotype 14 and its LipDH haplotype 19 with Tbr02 from Zambia and Tbb08 from Kenya, both members of the Kiboko group, suggesting relatively recent common ancestry. Note that Tev42 (KETRI2479), the lone representative of the ‘atypical’ LipDH genotype, is not represented in this network since this isolate lacks a maxicircle and therefore the COX1 gene.

### Principal component analysis (PCA) of microsatellite markers

**Figure 7:**
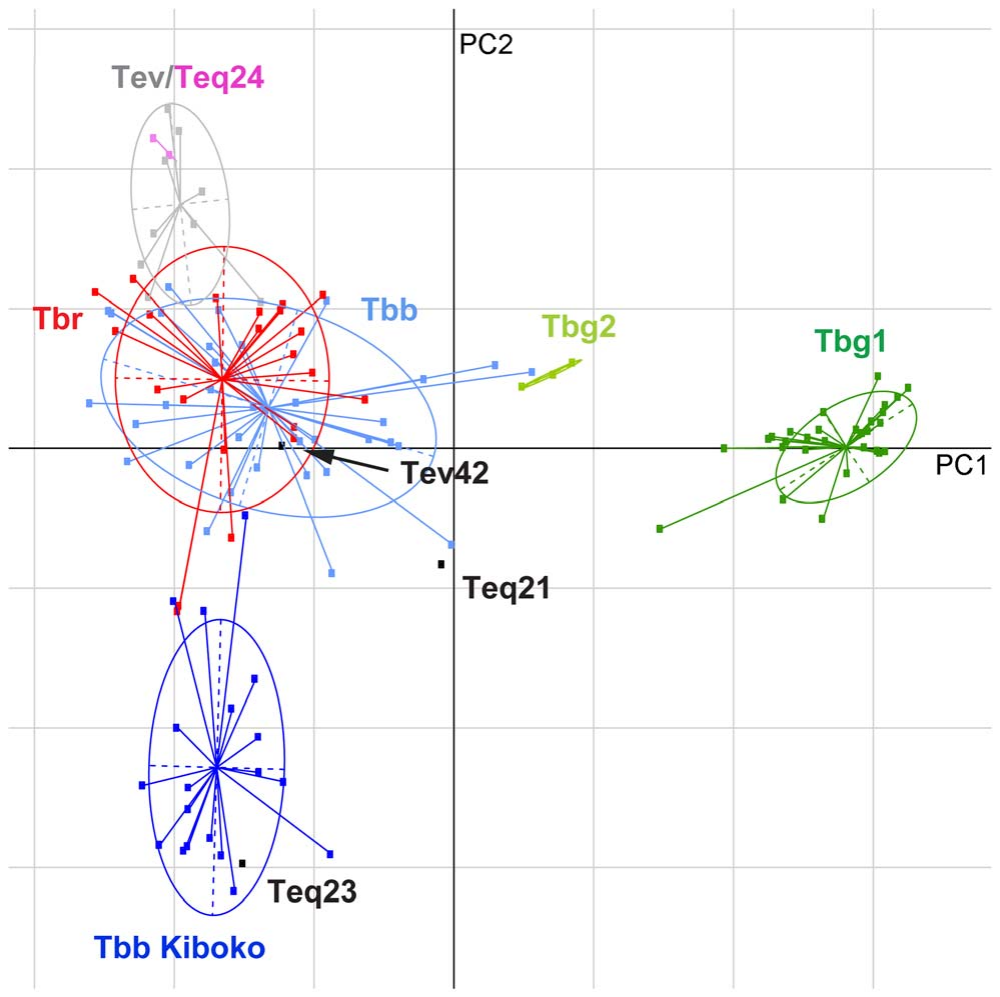
Evaluation of the genetic differentiation between isolates of *T. evansi* and *T. equiperdum* and subspecies of *T. brucei* using principal component analysis (PCA) of microsatellite data. PCA was performed in R using the package adegenet. Within subspecies of *T. brucei*, the differentiation between temporally and geographically cohesive subgroups was estimated using DEST and calculated with the program smogd. Points representing individual genotypes are connected by a line to the centroid of an ellipse, which circumscribes a region encompassing 95% of the variance observed within six trypanosome subgroups identified: major *T. evansi* and *T. equiperdum* group (grey and pink), *T. b. rhodesiense* (red), *T. b. brucei* Kiboko (dark blue), *T. b. brucei* non-Kiboko (light blue), *T. b. gambiense* group 1 (dark green), *T. b. gambiense* group 2 (light green). *T. evansi* and *T. equiperdum* isolates that fell outside the major group are shown as black data points. The wide distribution of *T. evansi* and *T. equiperdum* isolates among distinct subgroups strongly supports multiple independent origins for these dyskinetoplastic strains. The first two principal components (PC1 and PC2) explain 29.2% and 8.4% of the total variance in the data, respectively. Data for isolates other than *T. evansi* and *T. equiperdum* had been published previously [40].

Incorporation of microsatellite data for a subset of *T. evansi*/*T. equiperdum* isolates into an established PCA network [40] also gave results that are inconsistent with monophyly of either species (Fig. 7). Most *T. evansi* isolates, together with Teq24 (STIB818), formed a cluster (grey circle) related to, but somewhat distinct from, non-Kiboko *T. b. brucei* (light blue circle) and *T. b. rhodesiense* (red circle). The single exception among *T. evansi* was again Tev42 (KETRI2479), which localized near the centre of the non-Kiboko cluster. Teq21 (BoTat1.1) was also more related to non-Kiboko *T. b. brucei*, but relatively distant from Tev42. The PCA analysis, consistent with the COX1 and LipDH data, suggested a relatively close evolutionary relationship of *T. equiperdum* STIB841/OVI (Teq23) with the Kiboko group of *T. b. brucei* (dark blue circle).

The results of the phylogenetic analyses are summarized in Table 4, along with the ATPase subunit γ genotypes and, where known, dominant minicircle class and RoTat 1.2 VSG genotype. Combined, these markers suggest that the *T. evansi*/*T.equiperdum* isolates investigated in our study can be arranged into four distinct groups of independent evolutionary origin (see Discussion)

**Table 4:** Proposed grouping of *T. evansi*/*T. equiperdum* isolates.

Group	1	2	3	4
**Examples (code)**	*T. ev.* STIB805 (27)*T. ev.* Kazakh (33)*T. ev.* AnTat3/3 (36)*T. eq.* STIB818 (24)*T. eq.* ATCC30023 (25)	*T. eq.* STIB842 (22)*T. eq.* BoTat1.1 (21)	*T. eq.* STIB841^1^ (23)	*T. ev.* KETRI2479 (42)
**ATPase γ C-term.**	A281del	A273P	WT	M282L
**dominant minicircle class**	A^2^	C^3^	n/a	B^4^
**LipDH genotype**	W/Z	V/Z	V/V	atypical
**COX1 haplotype**	23	22	14	n/a^5^
**microsatellite PCA**	Tev	Tbb	Tbb Kiboko	Tbb
**RoTat1.2**	+^6^	-7	-7	-8

1 Probably identical or highly similar to *T. equiperdum* OVI ([5] and subsequent communications in *Trends in Parasitology*: http://www.cell.com/trends/parasitology/abstract/S1471-4922(05)00334-X)

2 [90]

3 [4]

4 [90], [91]

5 n/a  =  not available, as KETRI2479 lacks COX1

6 this work, [92]–[95]

7 [93]

8 [96]

## Discussion

We sequenced and analyzed the genome of *T. evansi* strain STIB805, a representative of a large, clonal group of close relatives of *T. brucei* that are transmitted independently of tsetse flies. Our examination revealed a number of insights into the biology and phylogenetic origin of this akinetoplastic trypanosome and provides further support for the proposed classification of *T. evansi* and *T. equiperdum* as subspecies of *T. brucei* [4]. A comparison with the *T. b. brucei* TREU 927/4 reference shows broad similarity, with 94.6% of non-repetitive CDS having a nucleotide identity of 95% or greater and 78.9% having ≥99% identity (Fig. 2). This high degree of identity mirrors similarities previously observed between *T. b. brucei* TREU 927/4 and *T. b. gambiense* DAL972 (86.4% of CDS had ≥99% identity) [33]. Phylogenetic analyses of *T. evansi* STIB805 VSG sequences also show extensive similarity to *T. b. brucei*. A cladistic representation of VSG sequences from *T. evansi* and *T. b. brucei* shows these sequences broadly interspersed with little evidence for subspecies-specific grouping, which underscores the close evolutionary relationship of the two strains (S10 Fig.). Despite the lack of kDNA and the absence of the life cycle stages in the insect vector, where the *T. b. brucei* mitochondrion is exclusively active in oxidative phosphorylation, *T. evansi* has maintained the coding capacity for nearly all of the mitochondrial proteome found in *T. b. brucei*. To some extent this reflects the fact that many mitochondrial activities are expected to remain essential even in an akinetoplastic bloodstream trypanosome. Such activities include the glycine cleavage complex [65], the alternative oxidase [66] and ubiquinone biosynthesis [67], fatty acid metabolism [68], [69], the F_1_-ATP synthase and ADP/ATP carrier [20], iron sulfur cluster biosynthesis [70], and all activities required to maintain and duplicate the mitochondrion itself. Nonetheless, a focused analysis of those genes known to be involved in kDNA maintenance or expression, or in oxidative phosphorylation, identified not a single case of gene loss and very few cases of mutations with predicted consequences for protein function. Of the 35 kDNA replication and transcription CDS examined, only the mitochondrial DNA polymerase beta-PAK appeared to be functionally compromised (S6 Table). Similarly, all 133 of the CDS identified as the mitochondrial ribosome in *T. b. brucei* [62], [71] are maintained in *T. evansi*, with the largest difference observed being a relatively small region of TevSTIB805.11_01.4800 (Tb927.11.4650) that alters 24 amino acids in the middle of the coding sequence. The paucity of disruptions among such a large protein complex strongly suggests that the mitochondrial ribosomes in *T. evansi* STIB805 are fully functional despite the lack of known substrates. Editosome function also appears essentially intact in *T. evansi*. Previous experiments have shown that dyskinetoplastic *T. b. brucei* and another strain of *T. evansi* contain functional editing complexes, which is consistent with retention of proteins no longer required [72].

Components of the mitochondrial electron transport chain are of particular interest in *T. evansi*, as the key compensatory change that permits survival in the absence of kDNA resides in complex V (mitochondrial F_O_F_1_-ATP synthase) [20]. The electron transport chain is composed of five complexes in the inner mitochondrial membrane, and in *T. brucei* these complexes are differentially expressed between life cycle stages [9]. In bloodstream forms, ATP is thought to be produced entirely by glycolysis. The classical respiratory chain is not functionally active, and the ATP synthase operates “in reverse” by hydrolyzing ATP to pump protons into the mitochondrial matrix in order to maintain the essential mitochondrial membrane potential, Δψm [19], [73], [74]. This activity requires the kDNA-encoded F_O_ subunit 6. Maintenance of Δψm in the absence of a functional F_O_ in *T. evansi* STIB805 involves an A281 deletion in the gamma subunit of the F_1_ subcomplex, but whether this mutation alone is sufficient to fully compensate for kDNA loss is presently unclear [20]. Our study has identified mutations in the α and β subunits of F_1_ that could function in concert with the A281 deletion to permit survival of *T. evansi* STIB805, and this can be tested experimentally. Nuclear genes encoding components of complexes I-IV of the oxidative phosphorylation system appear to be intact in *T. evansi*. Although complex I is expressed in bloodstream *T. brucei*, a recent study demonstrated that it is non-essential for bloodstream form metabolism, and no growth defects were observed in cell lines in which complex I components were eliminated [75]. Genes for subunits of complexes II-IV are not required in bloodstream form parasites, and eleven kDNA-encoded subunits are absent in *T. evansi*, yet the integrity of the nuclear-encoded genes for complexes I-IV has been maintained in *T. evansi* STIB805.

*Ka*/*Ks* analysis further highlights the conservation of the *T. evansi* mitoproteome in general and of a subset of proteins involved in kDNA maintenance, expression, and function in particular: we detected no significant difference between *Ka*, *Ks*, or *Ka*/*Ks* values for *T. evansi* STIB805 and *T. b. gambiense* DAL972 relative to *T. b. brucei*. A potential explanation is that selective pressure remains for a large number of the mitoproteome CDS, including those involved in kDNA maintenance and function, perhaps due to additional functions outside of the mitochondrion. For example, the mitochondrial topoisomerase II was recently shown to be important for normal growth even in the absence of kDNA [20]. Alternatively, retention of functional sequences could reflect a relatively short evolutionary separation between *T. brucei* and *T. evansi* STIB805, high stability of the gene maintenance and expression pathways in these organisms, or some unknown aspect of their biology. For example, robust mechanisms to maintain gene functionality would make sense in an organism with a complex life cycle like *T. brucei* that spends many generations in an environment where only a subset of genes are required.

The depth of similarity between *T. evansi* STIB805 and *T. b. brucei* TREU 927/4 creates a background that highlights some of the observed differences. The differences in chromosome pattern between the two *T. evansi* and *T. b. brucei* strains are comparable to strain variations observed within the *T. b. brucei* subspecies (Fig. 1) [46]. Previous studies investigating *T. brucei* chromosomes have indicated that gene content is more conserved than chromosome size [30], [76], similar to other kinetoplastid species [77], [78]. This size polymorphism is probably caused by genome-wide rearrangements over evolutionary time. The majority of the megabase chromosomes appeared largely similar between *T. evansi* STIB805 and *T. b. brucei* TREU 927/4, which is consistent with similarities subsequently observed in sequence. It remains to be shown whether the chromosomal differences observed among trypanosomatids reflect any functional consequences, which are more obvious at the gene level.

One clear example of such CDS differences that are found in the *T. evansi* STIB805 genome is the loss of a significant number of procyclin associated genes (PAGs). In multiple *T. brucei* strains, the chromosome 10 procyclin locus is heterozygous, with one chromosome containing EP1/EP2/PAG1/PAG5/PAG2*/PAG4 and the other chromosome containing EP1/EP2/PAG2/PAG4 [49], [52]–[56]. In *T. evansi* STIB805, chromosome 10 is homozygous for the EP1/EP2/PAG2/PAG4 locus, resulting in the associated absence of PAG5 and PAG2*. Also on chromosome 10, the EP2 gene in *T. evansi* contains a stretch of 12 divergent amino acids in the domain N-terminal to the EP repeat; this region is highly conserved among *T. b. brucei* strains [57], [58]. The PAG3 and GRESAG2 genes on chromosome 6 have frame-shifts and deletions. Although the function of procyclins and PAGs are not fully clear, the hypothesized role in protection within tsetse fly midgut is a logical extrapolation [50]. Because *T. evansi* has jettisoned this life-cycle stage, a life persisting as bloodstream parasites provides no selection to maintain these genes. Nonetheless, the fact that several procyclin genes and PAGs have been disrupted in *T. evansi* STIB805 stands in stark contrast to the integrity of other genes dispensable in an akinetoplastic bloodstream trypanosome, such as the genes specifically required for kDNA maintenance or expression, or for insect stage-specific energy metabolism (see above). Procyclin genes - in contrast to most other protein coding genes - are transcribed by RNA polymerase I, which might be associated with idiosyncratic features at these loci. Thus, either the loss of the genes gives a selective advantage to parasites locked in the bloodstream stage, or their genomic loci are intrinsically less stable.

Several other genes appear to be missing in *T. evansi* STIB805, but all cases we identified represent members of gene families that show considerable variation among *T. brucei* subspecies and strains. For example, presence of the complete IAO/AT2 array on *T. b. brucei* TREU 927/4 chromosome 2 and of the single IAO gene on chromosome 9 (Tb927.9.14600) is highly variable among *T. b. brucei* and *T. b. gambiense* strains [33]. Similarly, tandem duplications on *T. b. brucei* TREU 927/4 chromosomes 3 (Tb927.3.5690-.5730) and 6 (Tb927.6.1310-.1390) are absent from both STIB805 and *T. b. gambiense* DAL972 [33]. Hence, with the notable exception of procyclins and PAGs, most cases of genes that are present in Tb927 but absent from *T. evansi* STIB805 are probably more a reflection of frequent differences in gene content within the subgenus *Trypanozoon*, rather than events specific to *T. evansi*. It is unknown if this variability is of any biological significance.

A search for ORFs on contigs assembled *de novo* from *T. evansi* STIB805 Illumina reads identified 200 candidates for *T. evansi*-specific genes (S1 Data File). These candidates lacked close homologs in other trypanosome species based on the absence of BLASTp hits (E-value cut-off 0.001) in Tb927 or in the NCBI database (Table 2 and S2 Table). Seven of these candidates came from contigs with at least 5x minimal coverage and mapping of *T. b. brucei* TREU 927/4 or Lister 427 Illumina reads to these ORFs resulted in no or only incomplete coverage. The size of these ORFs is generally small (306–501 bp) and the surrounding contig regions either lack homology to Tb927 chromosomes entirely or match to repetitive regions such as VSG arrays. Further studies are needed to elucidate whether any of these candidates are functional CDS, and whether they are generally absent from *T. brucei* ssp. and present in *T. evansi* strains, and therefore are of potential diagnostic value. We confirmed the presence of the RoTat 1.2 gene in the STIB805 genome, which encodes a VSG used for serodiagnosis of surra [79]. Although the N-terminal domain is almost identical to the canonical RoTat 1.2 sequence (NCBI accession no. AF317914), the STIB805 protein has a different C-terminal domain. Based on our phylogenetic analysis we would caution that the RoTat 1.2 gene may not reliably distinguish surra from dourine (see below).

The relationship of *T. evansi* strains to each other as well as to strains classified as *T. equiperdum* and to the *T. brucei* group is controversial [5], [11], [80]. Hoare had suggested that *T. evansi* evolved from a *T. brucei* infection in camels that had temporarily entered the sub-Saharan tsetse belt. This strain subsequently became adapted to mechanical transmission by biting flies and disseminated by caravans to areas outside of sub-Saharan Africa. Hoare further speculated that the sexually transmitted *T. equiperdum* may have evolved from an equine strain of *T. evansi* by developing tropism for genital tissues [81]. Subsequent molecular analyses demonstrated that most strains of *T. equiperdum*, in contrast to *T. evansi*, possess at least a partial maxicircle (reviewed in [8]), which is inconsistent with a scenario in which the former evolved from the latter. Brun *et al*. proposed a reversed scenario in which *T. evansi* arose from a clone of *T. equiperdum* that had lost its maxicircle [3]. More extensive phylogenetic studies suggested that the *T. evansi*/*T. equiperdum* group is not monophyletic, leading to the hypothesis that some *T. equiperdum* isolates are misclassified *T. evansi* strains whereas others are a subspecies of *T. brucei* [5], [82]. A more recent phylogenetic analysis based on SL RNA repeats from various isolates of *T. evansi*, *T. equiperdum*, *T. b. brucei* and *T. b. gambiense* found no species-specific clusters, and the authors suggested that the various *T. evansi* and *T. equiperdum* strains may have evolved on numerous independent occasions and should be classified as subspecies of *T. brucei* [1], [4].

The phylogenetic analysis presented in the present study, which was based on four different genetic markers, strongly suggests that *T. evansi*/*T. equiperdum* evolved from within the *T. brucei* group on at least four independent occasions and from genetically distinct *T. brucei* strains (Table 4 and Fig. 5–7). Isolates within each group share not only the genotypes assessed in the present study, but also the dominant class of minicircle (if present) and the RoTat 1.2 genotype. The grouping presented in Table 4 is also consistent with a phylogeny based on random amplified polymorphic DNA and a multiple endonuclease typing approach [5], [82]. Not all markers listed in Table 4 have been assessed for all strains, but all the data available are consistent with this grouping, which provides a solid framework for further studies. The isolates we sampled cover a time period of 110 years and a vast geographical area (S7 Table), but it seems likely that other independent lineages of *T. evansi*/*T. equiperdum* have emerged earlier, and that independent extant lineages exist that were not sampled in the present study.

Another important observation from the phylogenetic data summarized in Table 4 is that Group 1 contains isolates representing both *T. evansi* and *T. equiperdum*. A similar intermingling of isolates classified as *T. evansi* and *T. equiperdum* was observed in other phylogenetic studies and led the authors to hypothesize that these *T. equiperdum* strains had been misclassified and are actually *T. evansi* [5]. Another possibility is that *T. evansi* can under certain circumstances evolve from *T. equiperdum* by converting from a tissue parasite back to a blood parasite, and from sexual transmission to mechanical transmission by biting flies, as suggested by Brun *et al*. [3]. It remains mysterious why sexual transmission in horses by and large appears to be correlated with the presence of a (sometimes partial) maxicircle, and mechanical transmission by biting flies with a lack thereof. Based on our current knowledge of kinetoplast function in trypanosomes the maxicircle becomes vestigial in the absence of minicircle heterogeneity, and apparent minicircle homogeneity is a hallmark of both *T. evansi* and *T. equiperdum* (reviewed in [8]). Finding answers to these important questions will require a more complete understanding of kinetoplast function, and a thorough molecular and epidemiological analysis of new *T. evansi* and *T. equiperdum* isolates.

Regardless of which of the above explanations for the simultaneous presence of *T. evansi* and *T. equiperdum* isolates in Group 1 is correct, it is evident that they both evolved independently on at least two distinct occasions, and neither is therefore monophyletic. This scenario is reminiscent of the situation with *T. b. gambiense*, which evolved from *T. b. brucei* on two independent occasions [40], [83]. An important difference is that *T. b. gambiense*, at least theoretically, can mate with *T. b. brucei* to form hybrids, and there is strong evidence that this occurs for group 2 *T. b. gambiense* [83]. In contrast, *T. evansi* and *T. equiperdum* can no longer develop in the tsetse vector, considered a requirement for mating, and are therefore now genetically isolated. A consistent solution to the species problem in trypanosomes, as in other organisms, has not been reached, and classification of species vs. (sub)species has often been subjective and utilitarian rather than logical [84],[85]. However, maintaining the rank of species for *T. evansi* and *T. equiperdum*, despite clear evidence for lack of monophyly in both cases, seems inconsistent even within the *Trypanozoon* group itself. We therefore recommend re-classification as subspecies, i. e. *T. b. evansi* and *T. b. equiperdum*.

The present study did not identify any obvious candidates for genetic changes that might underpin the switch from tsetse-dependent to mechanical transmission by biting flies in *T. evansi*. A mathematical model for mechanical transmission suggested a high level of parasitaemia in the blood as an important condition for its success [86]. *T. brucei* regulates its parasitaemia through a quorum sensing mechanism that results in density dependent differentiation into a non-proliferative ‘stumpy’ form that is pre-adapted to survival in the tsetse vector [87]. Adaptations in the signaling pathway responsible for this sensing could provide a mechanism for increased parasitaemia, and numerous components of this pathway have recently been identified [88]. Indeed, while stumpy forms were occasionally observed for *T. evansi*, they are usually absent [81]. However, an entirely uncontrolled proliferation in the blood would probably result in untimely death of the host, again detrimental to transmission. On the other hand, a multi-species parasite like *T. evansi* could have (i) an uncontrolled proliferation in some of its hosts, and thus be fatal, as in horses, and (ii) a more controlled proliferation in others, such as certain bovines, which may act as reservoir. It therefore seems plausible that successful adaptation to mechanical transmission requires striking a new balance between survival of parasite and host, resulting in a new equilibrium that involves a very large range of receptive hosts exhibiting variable susceptibility.

### Conclusions

Taken together, the genome analysis and accompanying phylogenetic studies presented in this work revealed important insights into the biology and evolution of *T. evansi* STIB805 and dyskinetoplastic trypanosomes in general. Their re-classification as subspecies of *T. brucei*, i. e. *T. b. evansi* and *T. b. equiperdum*, seems clearly justified considering the vast similarities observed at the genome level and the lack of monophyly confirmed by phylogenetic analyses. Important questions that remain include the molecular basis of tsetse-independent transmission and the evolutionary timescale of the appearance of the *T. evansi*/*T. equiperdum* groups. The genome data presented here provide an important tool for future studies aimed at resolving these questions.

## Supporting Information

S1 Fig **Log-scale frequency distribution of nucleotide identity between orthologous** ***T. evansi*** **STIB805 vs. Tb927 coding sequences, including pseudogenes.** This graph represents all 8421 total non-repetitive sequences analyzed.
(PDF)

S2 Fig **Homozygosity of the EP/PAG2 locus in** ***T. evansi*** **STIB805.** A: Differential RPKM plot. RPKM values for *T. b. brucei* TREU 927/4 and *T. evansi* STIB805 Illumina reads mapped to the Tb927 reference were normalized for average coverage and the log2 ratio Tb/Te determined. X-axis: distance from the left end of the chromosome in kbp. The central line indicates the mean of the off-set (median of the ratios). The plot also reflects the lack of putative ESAG3 genes Tb927.10.9460 and Tb927.10.9470 in STIB805 (see S2 Table). B: Alignment of a 14.9 kb *T. evansi* STIB805 *de novo* contig (bottom) to the procyclin locus on *T. b. brucei* TREU 927/4 chromosome 10 (top). The PAG genes on the bottom strand of chromosome 10 are shaded red. Red columns between contig and chromosome indicate homology. No contigs or reads corresponding to the PAG1-PAG5-PAG2* segment could be identified.
(PDF)

S3 Fig **Reduced coverage of the EP3/PAG3/GRESAG2 locus on chromosome 6.** Differential RPKM plot. RPKM values for *T. b. brucei* TREU 927/4 and *T. evansi* STIB805 Illumina reads mapped to the Tb927 reference were normalized for average coverage and the log2 ratio Tb/Te determined. X-axis: distance from the left end of the chromosome in kbp. The central line indicates the mean of the off-set (median of the ratios).
(PDF)

S4 Fig **Reduced coverage of the IAO/AT2 locus on chromosome 2.** A: Mapping of *T. evansi* STIB805 reads against the IAO/AT2 locus on chromosome 2 in Tb927. IAO genes are indicated by red asterisks. Reads that could be uniquely mapped to the reference are colored blue (paired), red (single forward) or green (single reverse). Reads that could be mapped to more than one position in the Tb927 reference were placed randomly and are colored yellow. B: Mapping of *T. b. brucei* TREU 927/4 Illumina reads to the Tb927 reference shown for comparison. C: Differential RPKM plot. RPKM values for *T. b. brucei* TREU 927/4 and *T. evansi* STIB805 Illumina reads mapped to the Tb927 reference were normalized for average coverage and the log2 ratio Tb/Te determined. X-axis: distance from the left end of the chromosome in kbp. The central line indicates the mean of the off-set (median of the ratios). Coding sequences for iron/ascorbate oxidoreductase (IAO) proteins are found on chromosomes 2 and 9 in the Tb927 genome, and both loci display differences between the *T. brucei* reference and *T. evansi* STIB805. The IAO genes on chromosome 2 alternate with adenosine transporter 2 (AT2) genes to form five tandem repeats, ending with an extra copy of the nucleoside transporter gene. Although a *de novo* contig spanning the region for the IAO/AT2 array on chromosome 2 is not available, the reference assembly indicates at least partial loss of the IAO genes in *T. evansi* STIB805. The genes on chromosome 2 are upregulated in cultured bloodstream form *T. brucei* compared to the procyclic stage [59] and four of these IAO CDS encode a putative peroxisomal targeting signal (PTS1). The closest relative to these IAO CDS in SwissProt is the fungal isopenicillin N synthase, which is a known peroxisomal protein (Fred Opperdoes, http://TriTrypDB.org Comment Id: 24680).
(PDF)

S5 Fig **Absence of IAO Tb927.9.16400 in** ***T. evansi*** **STIB805.** A: Differential RPKM plot. RPKM values for *T. b. brucei* TREU 927/4 and *T. evansi* STIB805 Illumina reads mapped to the Tb927 reference were normalized for average coverage and the log2 ratio Tb/Te determined. X-axis: distance from the left end of the chromosome in kbp. The central line indicates the mean of the off-set (median of the ratios). The plot also indicates differences in the SL RNA and bloodstream alanine-rich protein (BARP) arrays between the reference and *T. evansi* STIB805. B: Alignment of a 6.2 kb *T. evansi* STIB805 *de novo* contig to the Tb927.9.14600 region on *T. b. brucei* TREU 927/4 chromosome 9. Note that Tb927.9.14600 (yellow box) is flanked by a direct repeat, as indicated by the overlap between the two regions of homology. No CDS corresponding to the IAO on *T. b. brucei* chromosome 9 (Tb927.9.14600) could be found in *T. evansi* STIB805, and a 6.2 kb *de novo* contig spanning that region supports the absence of that gene. Analysis of the *T. b. gambiense* DAL972 genome similarly found that Tb927.9.14600 is absent from its genomic repertoire, and it is also absent from numerous other *T. brucei* strains [33]. To our knowledge, the function of the IAO genes is presently unknown.
(PDF)

S6 Fig **The repeat of Tb927.9.7950/Tb927.9.7960-related genes is absent in** ***T. evansi*** **STIB805.** Differential RPKM plot for the region surrounding the Tb927.9.7950/Tb927.9.7960-related genes on chromosome 9 in Tb927. RPKM values for *T. b. brucei* TREU 927/4 and *T. evansi* STIB805 Illumina reads mapped to the Tb927 reference were normalized for average coverage and the log2 ratio Tb/Te determined. X-axis: distance from the left end of the chromosome in kbp. The central line indicates the mean of the off-set (median of the ratios). Chromosome 9 in Tb927 contains a 5x repeat of genes related to a tandem of hypothetical genes. Our coverage analysis suggests that only one copy of the Tb927.9.7950/Tb927.9.7960 tandem is present in STIB805, similar to what has been reported for *T. b. gambiense* DAL972 [33]. Other cases where single copy genes have undergone duplication in *T. b. brucei* TREU 927/4, but not in *T. b. gambiense* DAL972 or *T. evansi* STIB805, are Tb927.3.5690-.5730 and Tb927.6.1310-.1390 [33].
(PDF)

S7 Fig **The repeat of Tb927.4.3200/Tb927.4.3210-related genes is absent in** ***T. evansi*** **STIB805.** A: Mapping of *T. evansi* STIB805 reads against the region of the Tb927.4.3200/Tb927.4.3210 repeat on chromosome 4 in Tb927_v4. Reads that could be uniquely mapped to the reference are colored blue, red or green. Reads that could be mapped to more than one position in the Tb927_v4 reference were placed randomly and are colored yellow. B: Mapping of *T. brucei* TREU 927 Illumina reads to the Tb927 reference shown for comparison. C: Differential RPKM plot. RPKM values for *T. b. brucei* TREU 927/4 and *T. evansi* STIB805 Illumina reads mapped to the Tb927 reference were normalized for average coverage and the log2 ratio Tb/Te determined. X-axis: distance from the left end of the chromosome in kbp. The central line indicates the mean of the off-set (median of the ratios). D: Alignment of a 60.7 kb *T. evansi* STIB805 *de novo* contig to the Tb927.4.3200/Tb927.4.3210 repeat region. Inversions are indicated in blue. Chromosome 4 in Tb927 contains a 4x repeat of a gene tandem for a hypothetical (Tb927.4.3200) and an ESAG11-related protein (Tb927.4.3210). This entire repeat appears to be largely missing or disrupted in STIB805. The downstream region may be inverted compared to Tb927, although we cannot rule out an assembly artefact caused by tRNA genes that flank the affected region (See panel D). In the TriTrypDB database (http://TriTrypDB.org), only four of these genes are annotated for *T. b. gambiense* DAL972 and none for *T. b. brucei* Lister 427. This repeat region may therefore show considerable variation among the *Trypanozoon* strains.
(PDF)

S8 Fig **Absence of Tb927.8.490 and Tb927.8.500 in** ***T. evansi*** **STIB805.** A: Mapping of *T. evansi* STIB805 reads against the region surrounding Tb927.8.490 and Tb927.8.500 in Tb927. Reads that could be uniquely mapped to the reference are colored blue, red or green. Reads that could be mapped to more than one position in the Tb927 reference were placed randomly and are colored yellow. B: Mapping of *T. b. brucei* TREU 927/4 Illumina reads to the Tb927 reference shown for comparison. C: Differential RPKM plot. RPKM values for *T. b. brucei* TREU 927/4 and *T. evansi* STIB805 Illumina reads mapped to the Tb927 reference were normalized for average coverage and the log2 ratio Tb/Te determined. X-axis: distance from the left end of the chromosome in kbp. The central line indicates the mean of the off-set (median of the ratios). D: Alignment of a 8.1 kb *T. evansi* STIB805 *de novo* contig to the Tb927.8.490/Tb927.8.500 region. These two genes for hypothetical proteins of unknown function are related to Tb927.8.510 and Tb927.8.520, respectively, and therefore probably the result of segmental duplication followed by diversification. Orthologs for these genes are annotated in TriTrypDB for *T. b. brucei* Lister 427, but not for *T. b. gambiense* DAL972.
(PDF)

S9 Fig **Absence of Tb927.8.7300 -.7330 in** ***T. evansi*** **STIB805.** A: Mapping of *T. evansi* STIB805 reads against the Tb927.8.7300 -.7330 region in Tb927. Reads that could be uniquely mapped to the reference are colored blue, red or green. Reads that could be mapped to more than one position in the Tb927 reference were placed randomly and are colored yellow. B: Mapping of *T. b. brucei* TREU 927/4 Illumina reads to the Tb927 reference shown for comparison. C: Differential RPKM plot. RPKM values for *T. b. brucei* TREU 927/4 and *T. evansi* STIB805 Illumina reads mapped to the Tb927 reference were normalized for average coverage and the log2 ratio Tb/Te determined. X-axis: distance from the left end of the chromosome in kbp. The central line indicates the mean of the off-set (median of the ratios). The plot also reflects the internal deletion in gene Tb927.8.7260 (see D). D: Alignment of a 40.4 kb *T. evansi* STIB805 *de novo* contig to the Tb927.8.7300 -.7330 region on chromosome 8 of Tb927. Note also the deletion in the mid region of Tb927.8.7260 (upstream of Tb927.8.7300) that results in a frame-shift and predicted truncation of the encoded protein. Interestingly, this gene is annotated as “kinetoplast-associated” in TriTrypDB. Chromosome 8 in Tb927 contains a tandem repeat of two VSG-related (Tb927.8.7300/Tb927.8.7320) and two hypothetical genes (Tb927.8.7310/Tb927.8.7330). The former have been reported to be important for growth of bloodstream form *T. b. brucei* Lister 427 [89]. All four genes are absent in STIB805 and, according to TriTrypDB, from *T. b. gambiense* DAL972 as well.
(PDF)

S10 Fig **Neighbour-joining molecular cladograms of a-type and b-type VSG, with terminal nodes labeled as** ***T. b. brucei*** **TREU 927/4 (black) and** ***T. evansi*** **STIB805 (red).** A. Cladogram showing the four recognized subgroups of a-type VSG (N1-3 and 5), estimated from a multiple protein sequence alignment of 470 characters using JTT matrix. B. Cladogram showing b-type VSG (N4) estimated from a multiple protein sequence alignment of 492 characters using JTT rate matrix. Both trees highlight the fact that *T. evansi* sequences are distributed throughout the *T. brucei* VSG tree without strain-specific clades.
(PDF)

S11 Fig **Identification of a RoTat1.2 ortholog in** ***T. evansi*** **STIB805.** A: Sequence alignment of bp 1-850 of an ORF on *T. evansi* STIB805 *de novo* contig 5566 with the *T. evansi* RoTat 1.2 gene (NCBI entry AF317914). B: Alignment of bp 851-1431 and 851-1412, respectively, of the same sequences. C: Alignment of bp 1223–1431 of contig 5566 ORF with bp 1138–1355 of the degenerate VSG Tb927.8.240.
(PDF)

S1 Data File **A non-redundant list of ORFs from** ***T. evansi*** **STIB805** ***de novo*** **contigs that did not have a BLASTx hit in NCBI database.**
(FAS)

S2 Data File **A list of** ***T. evansi*** **STIB805 a-type VSG sequences.**
(FAS)

S3 Data File **A list of** ***T. evansi*** **STIB805 b-type VSG sequences.**
(FAS)

S1 Table **A spreadsheet showing identified orthologs between Tb927 and** ***T. evansi*** **STIB805.**
(XLSX)

S2 Table **A spreadsheet showing CDS identified in** ***T. evansi*** **STIB805** ***de novo*** **assemblies.**
(XLSX)

S3 Table **A spreadsheet showing 36046 SNPs identified in non-repetitive CDS in** ***T. evansi*** **STIB805.**
(XLSX)

S4 Table **A spreadsheet showing 2297 indels identified in non-repetitive CDS in** ***T. evansi*** **STIB805.**
(XLSX)

S5 Table **A spreadsheet showing non-synonymous SNPs and indels in Illumina sequences from** ***T. b. brucei*** **TREU 927/4 and** ***T. evansi*** **STIB805 compared to Tb927.**
(XLSX)

S6 Table **A spreadsheet showing detailed analyses of sequences predicted to comprise the** ***T. evansi*** **STIB805 mitochondrial proteome, including proteins related to kDNA, editosome, Complexes I-V, and mitochondrial translation.**
(XLSX)

S7 Table **A spreadsheet showing** ***Trypanozoon*** **strains and microsatellite information.**
(XLSX)
