# Supplementary figures and images for: Genome and Phylogenetic Analyses of Trypanosoma evansi Reveal Extensive Similarity to T. brucei and Multiple Independent Origins for Dyskinetoplasty
Source: PLoS Negl Trop Dis. 2015 Jan 8;9(1):e3404. doi: 10.1371/journal.pntd.0003404 (PMC4288722; doi:10.1371/journal.pntd.0003404)

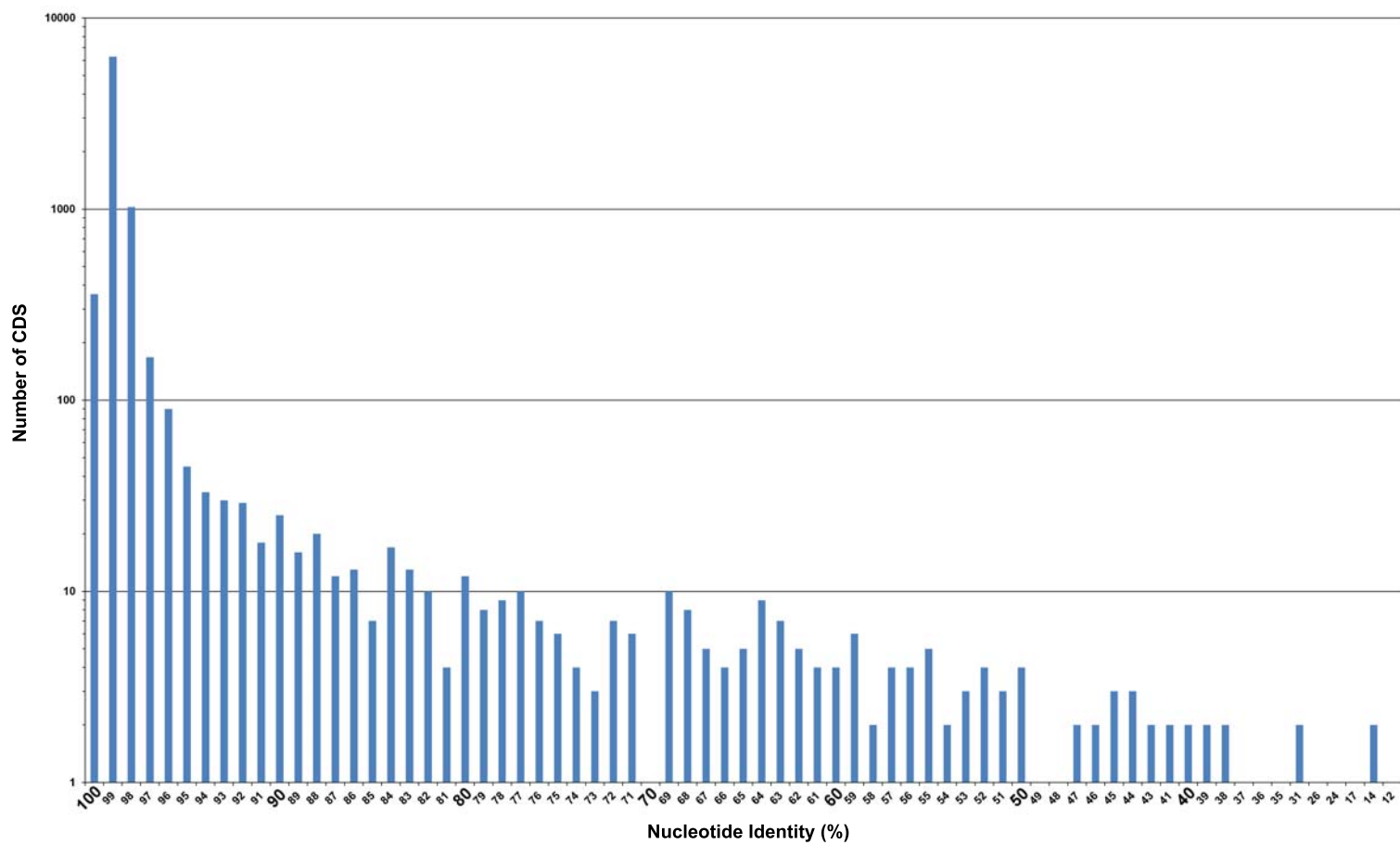

Supplement: S1 Fig — Log-scale frequency distribution of nucleotide identity between orthologous T. evansi STIB805 vs. Tb927 coding sequences, including pseudogenes. This graph represents all 8421 total non-repetitive sequences analyzed. (PDF) [file pntd.0003404.s001.pdf]

A

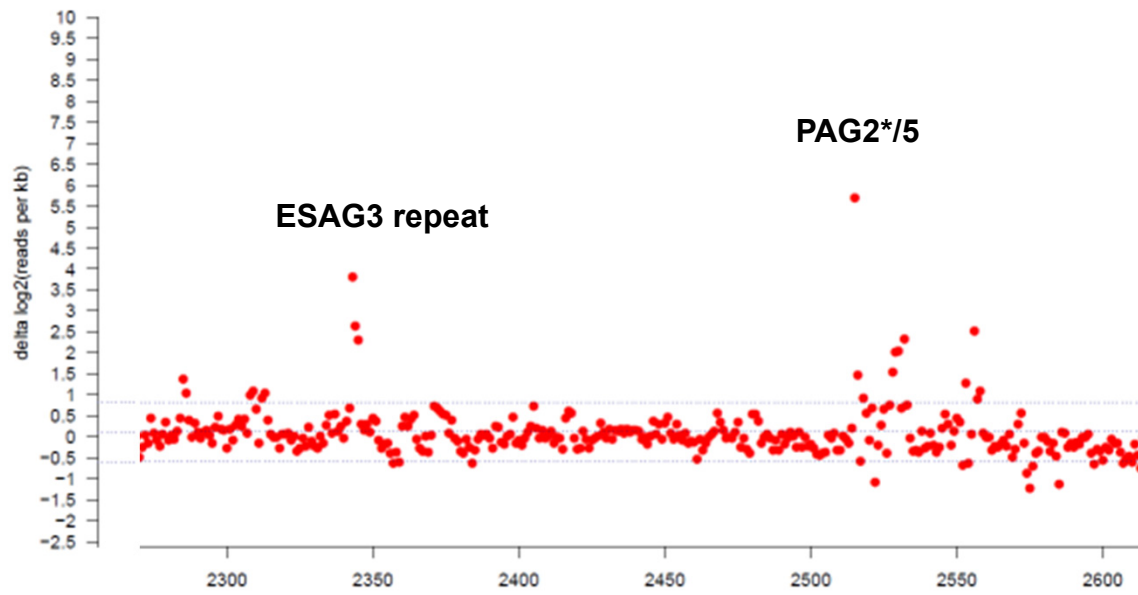

B

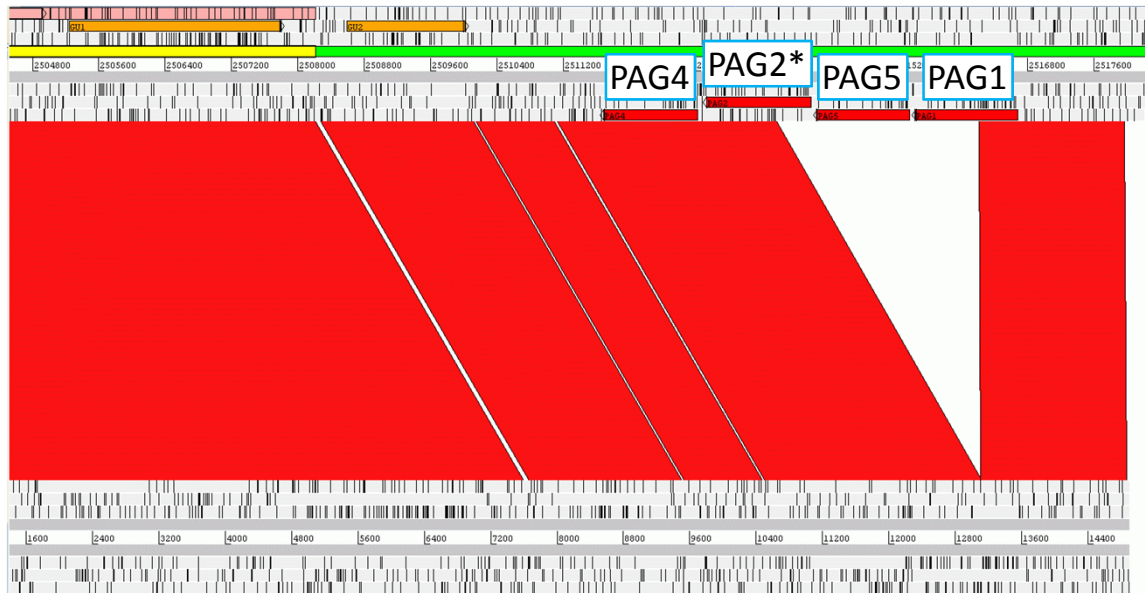

*T. evansi* STIB805 de novo contig

Supplement: S2 Fig — Homozygosity of the EP/PAG2 locus in T. evansi STIB805. A: Differential RPKM plot. RPKM values for T. b. brucei TREU 927/4 and T. evansi STIB805 Illumina reads mapped to the Tb927 reference were normalized for average coverage and the log2 ratio Tb/Te determined. X-axis: distance from the left end of the chromosome in kbp. The central line indicates the mean of the off-set (median of the ratios). The plot also reflects the lack of putative ESAG3 genes Tb927.10.9460 and Tb927.10.9470 in STIB805 (see S2 Table). B: Alignment of a 14.9 kb T. evansi STIB805 de novo contig (bottom) to the procyclin locus on T. b. brucei TREU 927/4 chromosome 10 (top). The PAG genes on the bottom strand of chromosome 10 are shaded red. Red columns between contig and chromosome indicate homology. No contigs or reads corresponding to the PAG1-PAG5-PAG2* segment could be identified. (PDF) [file pntd.0003404.s002.pdf]

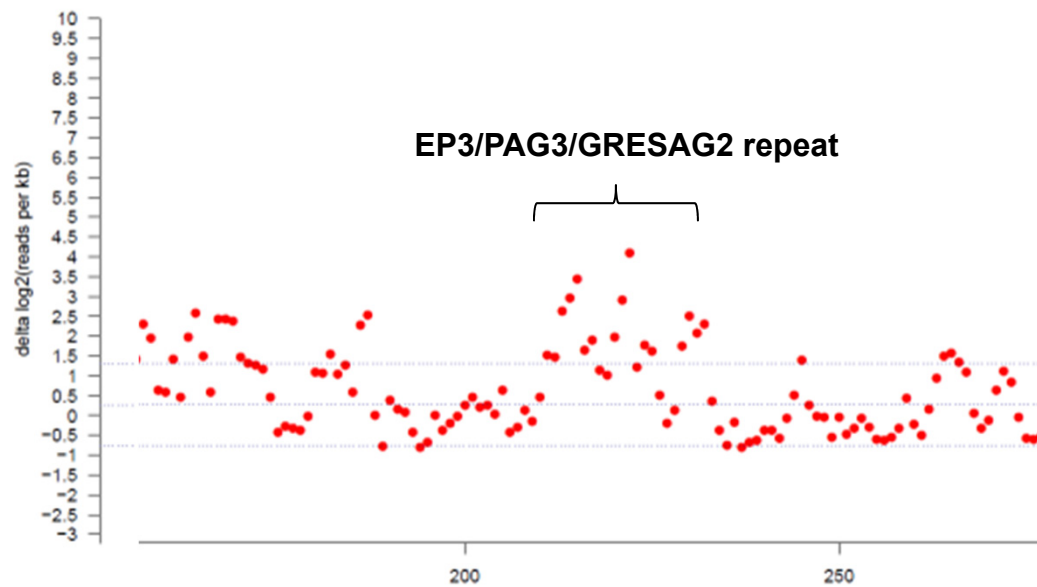

Supplement: S3 Fig — Reduced coverage of the EP3/PAG3/GRESAG2 locus on chromosome 6. Differential RPKM plot. RPKM values for T. b. brucei TREU 927/4 and T. evansi STIB805 Illumina reads mapped to the Tb927 reference were normalized for average coverage and the log2 ratio Tb/Te determined. X-axis: distance from the left end of the chromosome in kbp. The central line indicates the mean of the off-set (median of the ratios). (PDF) [file pntd.0003404.s003.pdf]

A

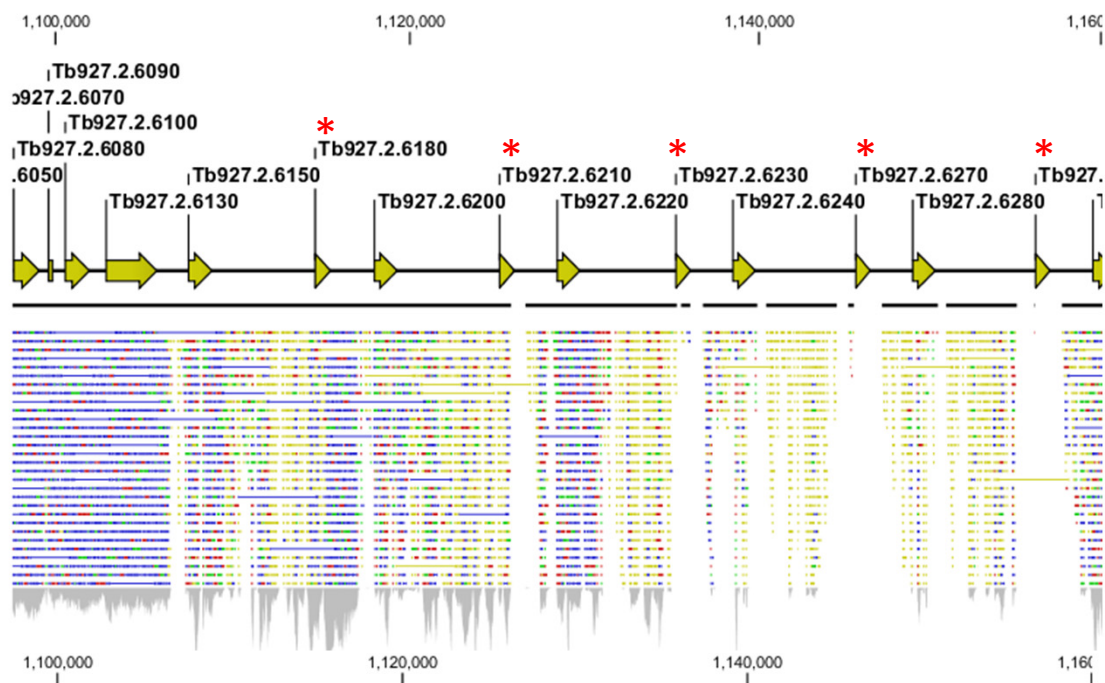

B

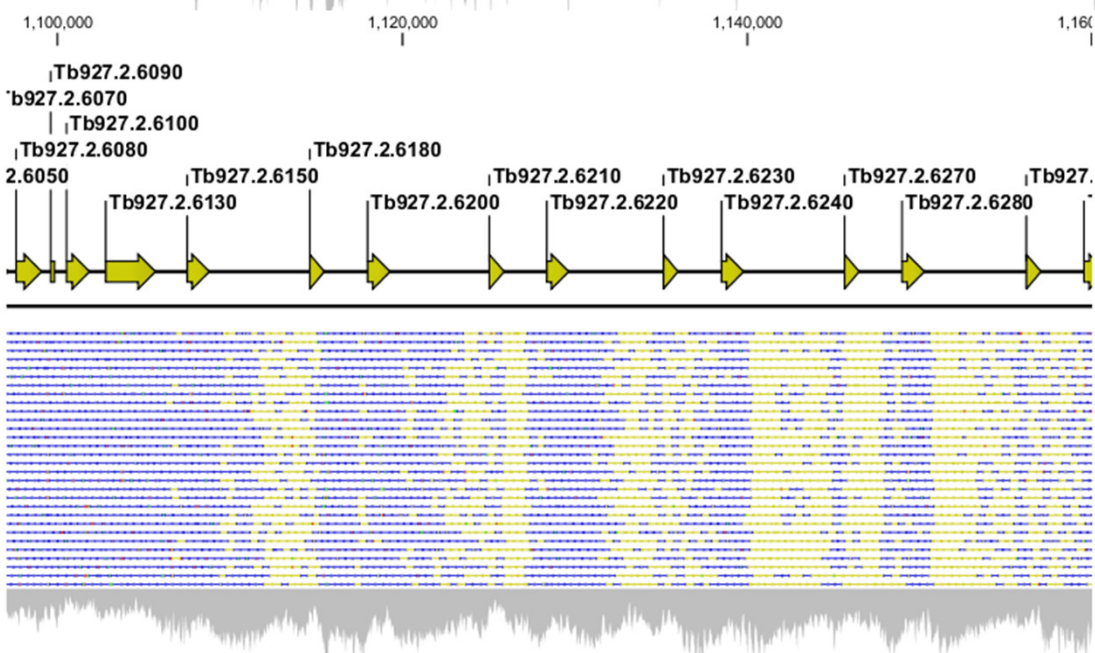

C

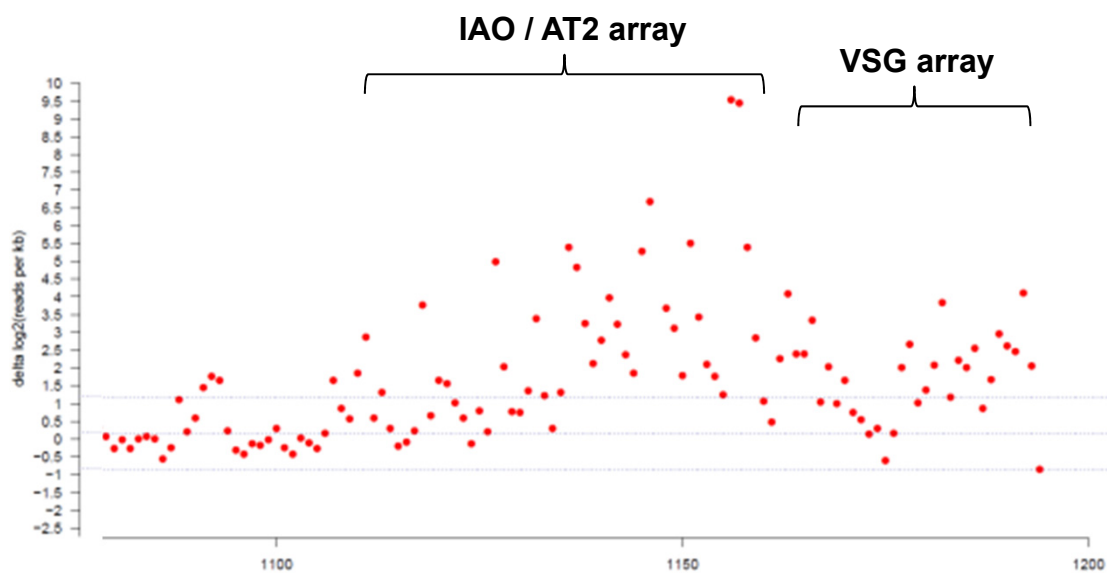

Supplement: S4 Fig — Reduced coverage of the IAO/AT2 locus on chromosome 2. A: Mapping of T. evansi STIB805 reads against the IAO/AT2 locus on chromosome 2 in Tb927. IAO genes are indicated by red asterisks. Reads that could be uniquely mapped to the reference are colored blue (paired), red (single forward) or green (single reverse). Reads that could be mapped to more than one position in the Tb927 reference were placed randomly and are colored yellow. B: Mapping of T. b. brucei TREU 927/4 Illumina reads to the Tb927 reference shown for comparison. C: Differential RPKM plot. RPKM values for T. b. brucei TREU 927/4 and T. evansi STIB805 Illumina reads mapped to the Tb927 reference were normalized for average coverage and the log2 ratio Tb/Te determined. X-axis: distance from the left end of the chromosome in kbp. The central line indicates the mean of the off-set (median of the ratios). Coding sequences for iron/ascorbate oxidoreductase (IAO) proteins are found on chromosomes 2 and 9 in the Tb927 genome, and both loci display differences between the T. brucei reference and T. evansi STIB805. The IAO genes on chromosome 2 alternate with adenosine transporter 2 (AT2) genes to form five tandem repeats, ending with an extra copy of the nucleoside transporter gene. Although a de novo contig spanning the region for the IAO/AT2 array on chromosome 2 is not available, the reference assembly indicates at least partial loss of the IAO genes in T. evansi STIB805. The genes on chromosome 2 are upregulated in cultured bloodstream form T. brucei compared to the procyclic stage [59] and four of these IAO CDS encode a putative peroxisomal targeting signal (PTS1). The closest relative to these IAO CDS in SwissProt is the fungal isopenicillin N synthase, which is a known peroxisomal protein (Fred Opperdoes, http://TriTrypDB.org Comment Id: 24680). (PDF) [file pntd.0003404.s004.pdf]

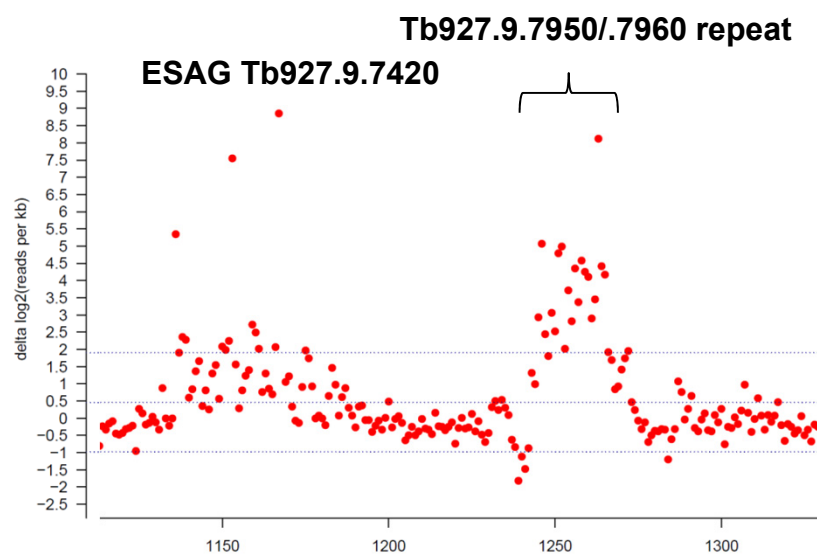

Supplement: S6 Fig — The repeat of Tb927.9.7950/Tb927.9.7960-related genes is absent in T. evansi STIB805. Differential RPKM plot for the region surrounding the Tb927.9.7950/Tb927.9.7960-related genes on chromosome 9 in Tb927. RPKM values for T. b. brucei TREU 927/4 and T. evansi STIB805 Illumina reads mapped to the Tb927 reference were normalized for average coverage and the log2 ratio Tb/Te determined. X-axis: distance from the left end of the chromosome in kbp. The central line indicates the mean of the off-set (median of the ratios). Chromosome 9 in Tb927 contains a 5x repeat of genes related to a tandem of hypothetical genes. Our coverage analysis suggests that only one copy of the Tb927.9.7950/Tb927.9.7960 tandem is present in STIB805, similar to what has been reported for T. b. gambiense DAL972 [33]. Other cases where single copy genes have undergone duplication in T. b. brucei TREU 927/4, but not in T. b. gambiense DAL972 or T. evansi STIB805, are Tb927.3.5690-.5730 and Tb927.6.1310-.1390 [33]. (PDF) [file pntd.0003404.s006.pdf]

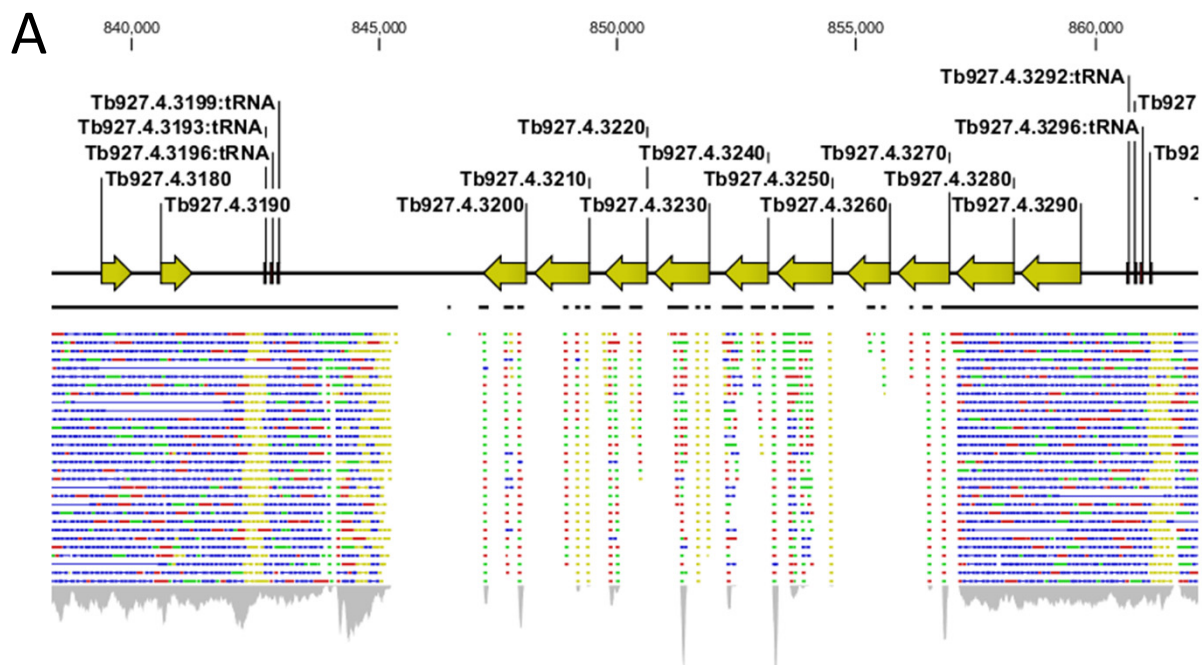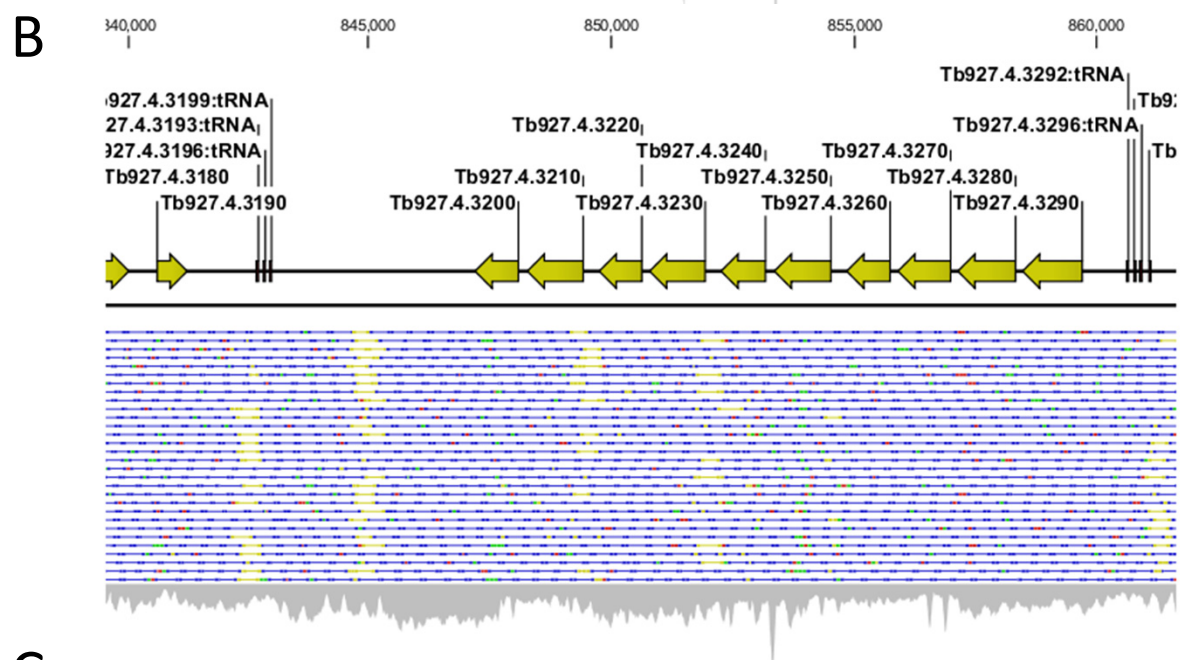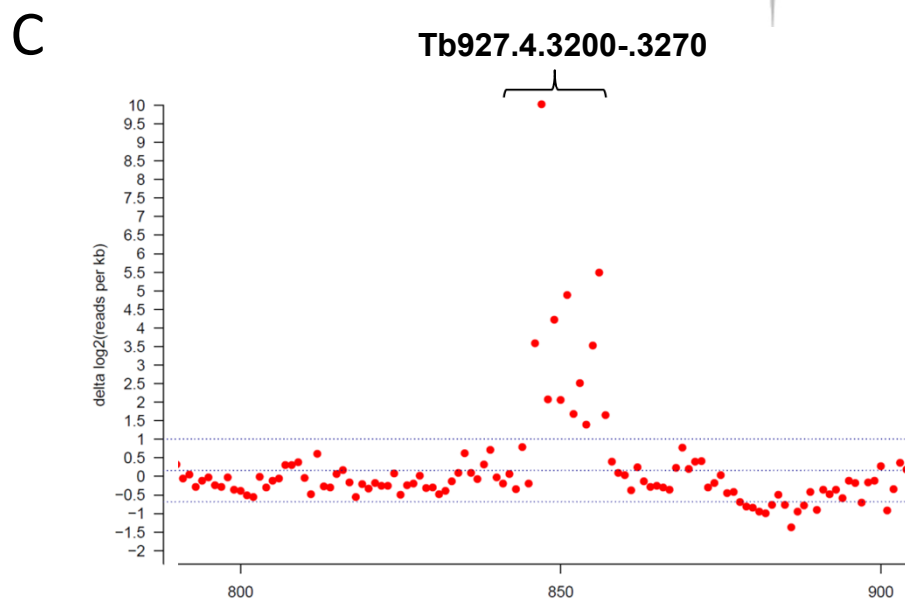

D

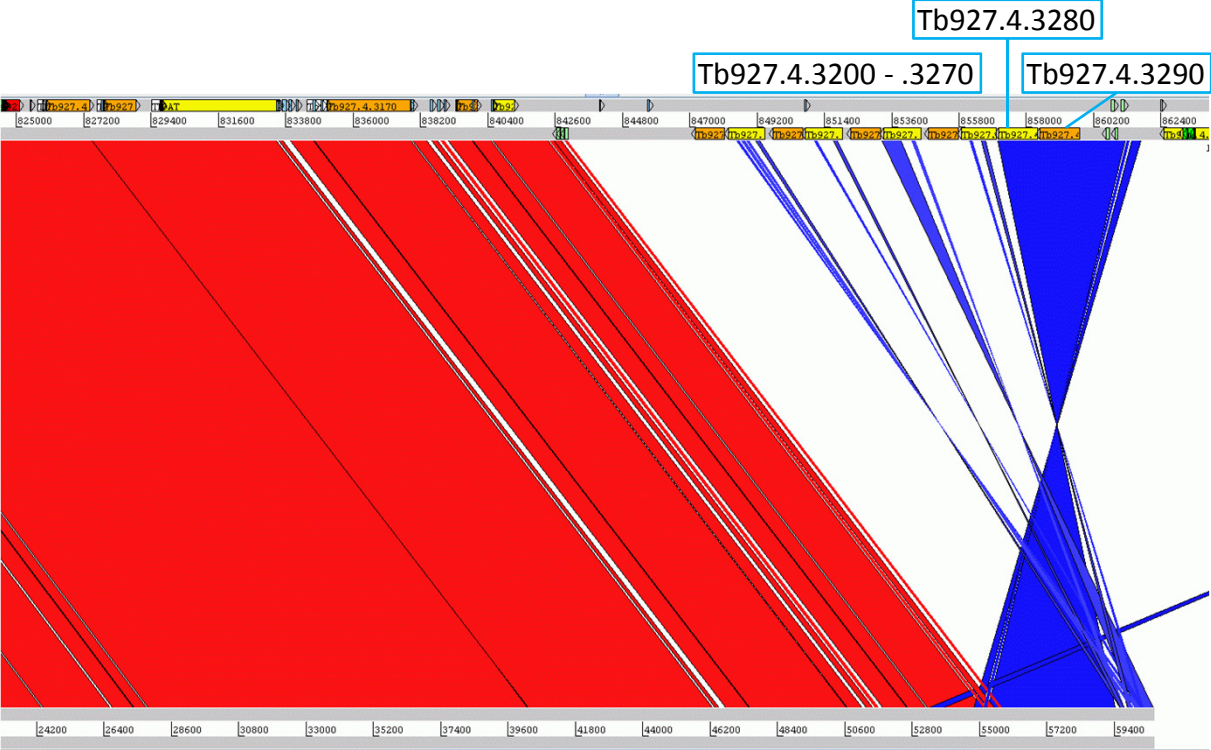

*T. evansi* STIB805 de novo contig

Supplement: S7 Fig — The repeat of Tb927.4.3200/Tb927.4.3210-related genes is absent in T. evansi STIB805. A: Mapping of T. evansi STIB805 reads against the region of the Tb927.4.3200/Tb927.4.3210 repeat on chromosome 4 in Tb927_v4. Reads that could be uniquely mapped to the reference are colored blue, red or green. Reads that could be mapped to more than one position in the Tb927_v4 reference were placed randomly and are colored yellow. B: Mapping of T. brucei TREU 927 Illumina reads to the Tb927 reference shown for comparison. C: Differential RPKM plot. RPKM values for T. b. brucei TREU 927/4 and T. evansi STIB805 Illumina reads mapped to the Tb927 reference were normalized for average coverage and the log2 ratio Tb/Te determined. X-axis: distance from the left end of the chromosome in kbp. The central line indicates the mean of the off-set (median of the ratios). D: Alignment of a 60.7 kb T. evansi STIB805 de novo contig to the Tb927.4.3200/Tb927.4.3210 repeat region. Inversions are indicated in blue. Chromosome 4 in Tb927 contains a 4x repeat of a gene tandem for a hypothetical (Tb927.4.3200) and an ESAG11-related protein (Tb927.4.3210). This entire repeat appears to be largely missing or disrupted in STIB805. The downstream region may be inverted compared to Tb927, although we cannot rule out an assembly artefact caused by tRNA genes that flank the affected region (See panel D). In the TriTrypDB database (http://TriTrypDB.org), only four of these genes are annotated for T. b. gambiense DAL972 and none for T. b. brucei Lister 427. This repeat region may therefore show considerable variation among the Trypanozoon strains. (PDF) [file pntd.0003404.s007.pdf]

A

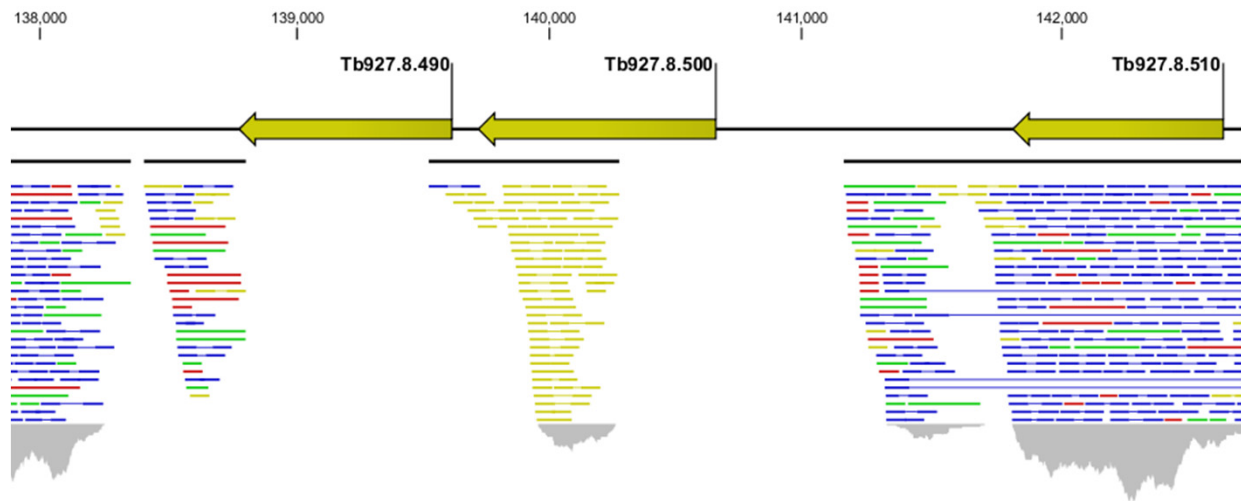

B

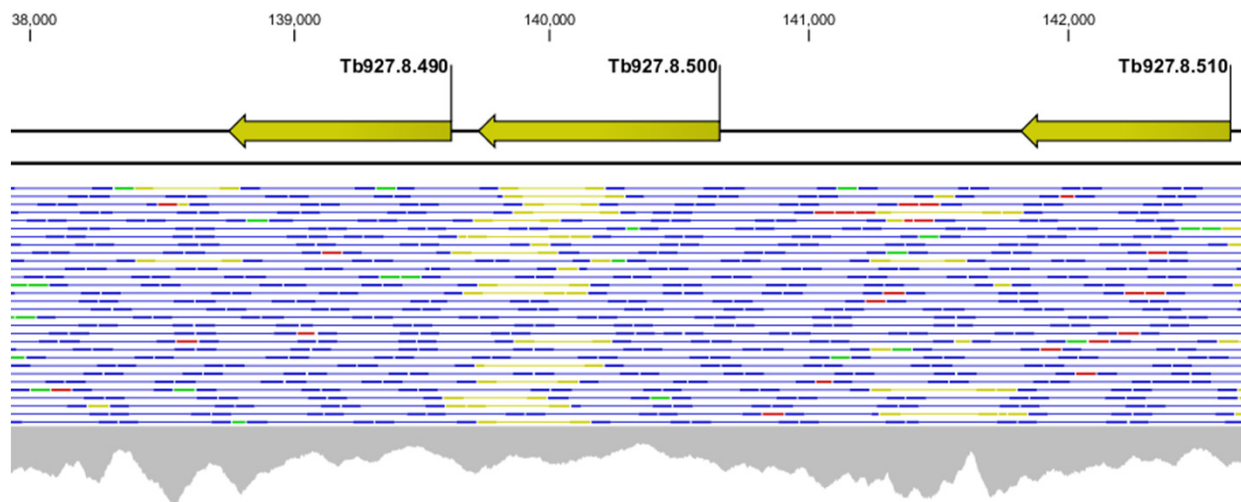

C

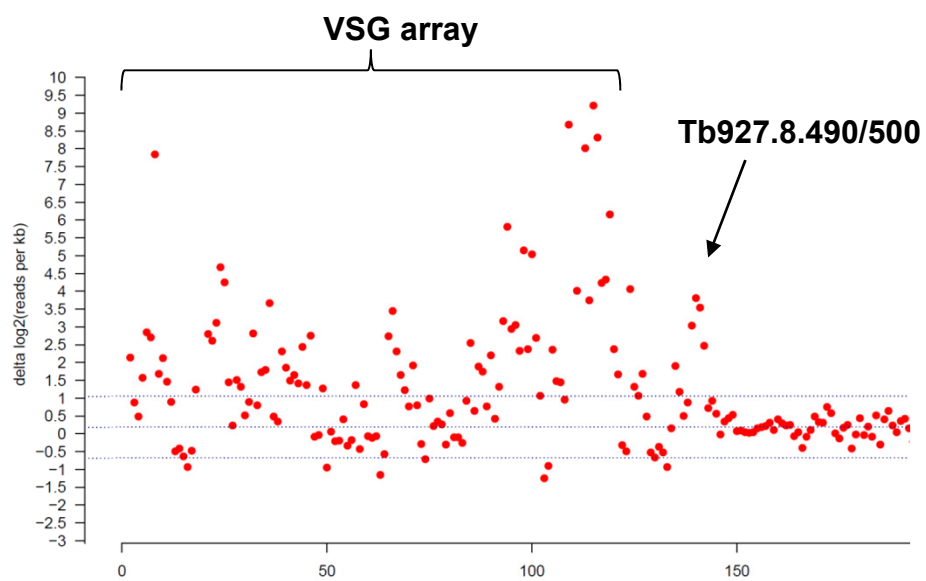

D

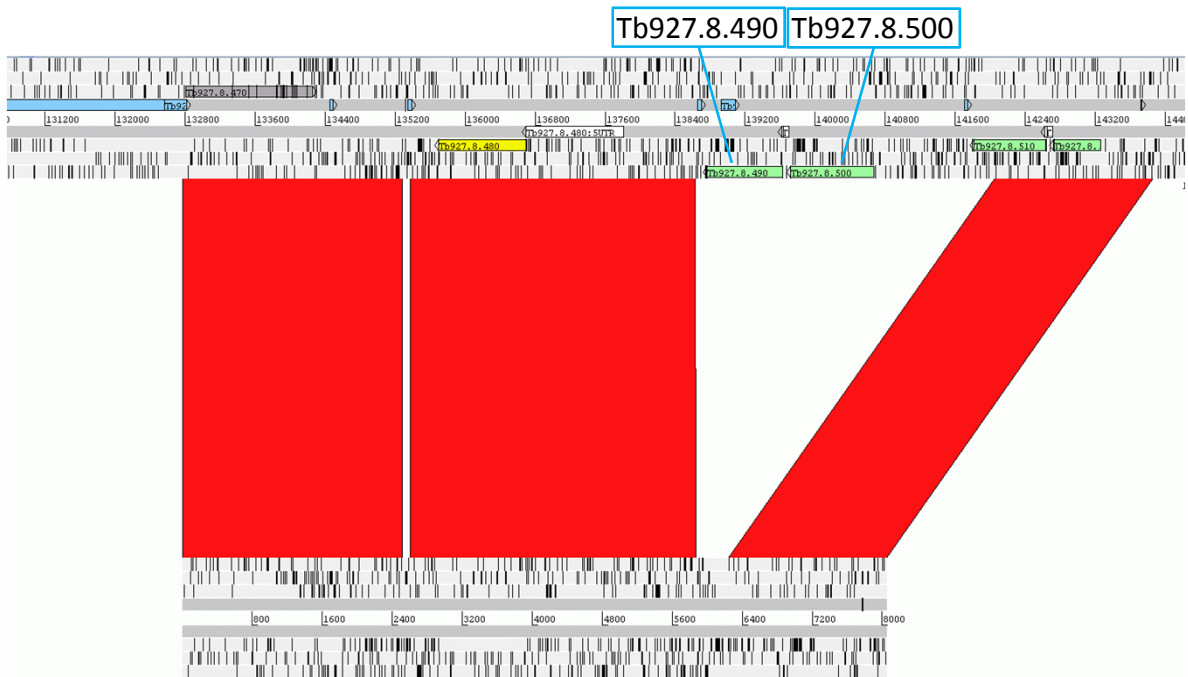

*T. evansi* STIB805 de novo contig

Supplement: S8 Fig — Absence of Tb927.8.490 and Tb927.8.500 in T. evansi STIB805. A: Mapping of T. evansi STIB805 reads against the region surrounding Tb927.8.490 and Tb927.8.500 in Tb927. Reads that could be uniquely mapped to the reference are colored blue, red or green. Reads that could be mapped to more than one position in the Tb927 reference were placed randomly and are colored yellow. B: Mapping of T. b. brucei TREU 927/4 Illumina reads to the Tb927 reference shown for comparison. C: Differential RPKM plot. RPKM values for T. b. brucei TREU 927/4 and T. evansi STIB805 Illumina reads mapped to the Tb927 reference were normalized for average coverage and the log2 ratio Tb/Te determined. X-axis: distance from the left end of the chromosome in kbp. The central line indicates the mean of the off-set (median of the ratios). D: Alignment of a 8.1 kb T. evansi STIB805 de novo contig to the Tb927.8.490/Tb927.8.500 region. These two genes for hypothetical proteins of unknown function are related to Tb927.8.510 and Tb927.8.520, respectively, and therefore probably the result of segmental duplication followed by diversification. Orthologs for these genes are annotated in TriTrypDB for T. b. brucei Lister 427, but not for T. b. gambiense DAL972. (PDF) [file pntd.0003404.s008.pdf]

A

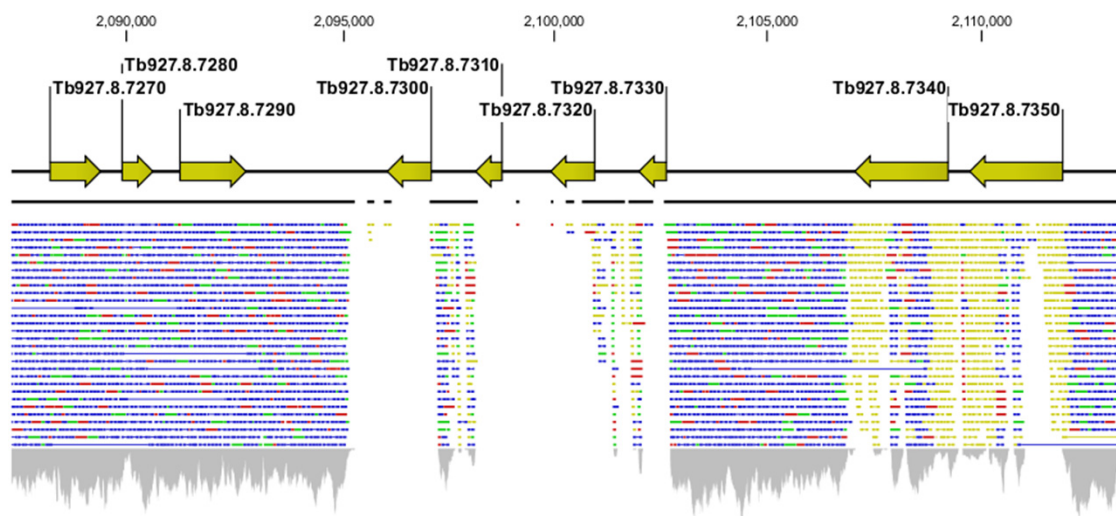

B

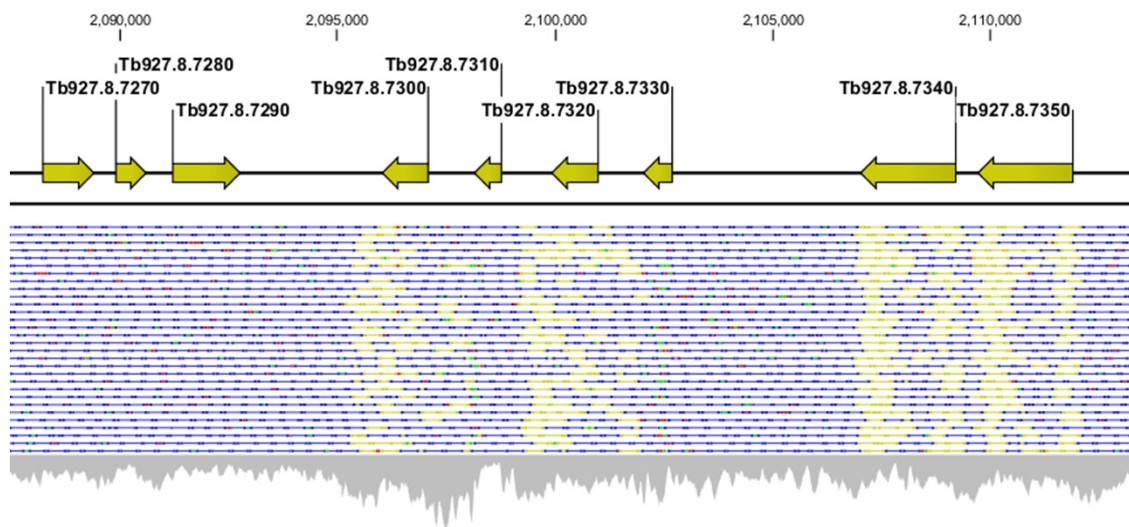

C

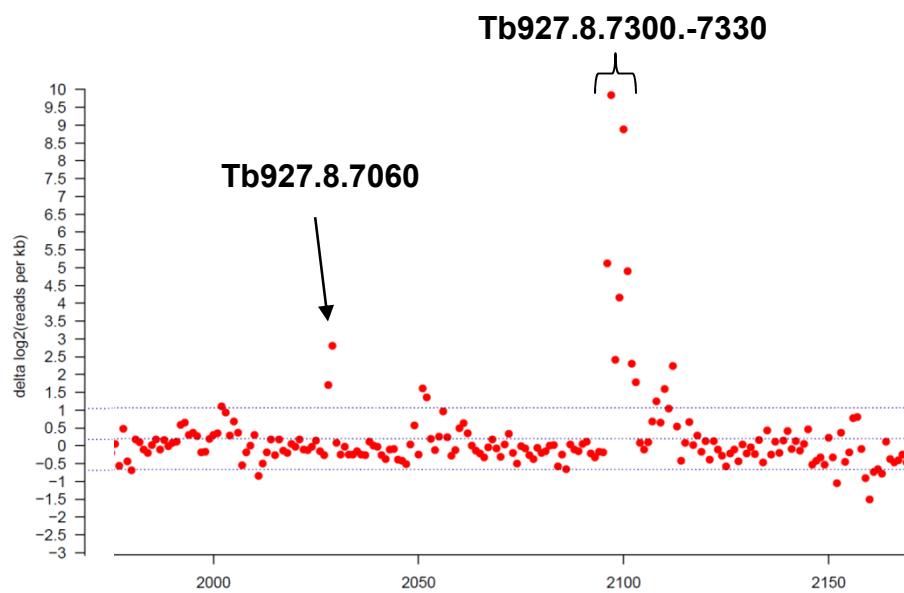

D

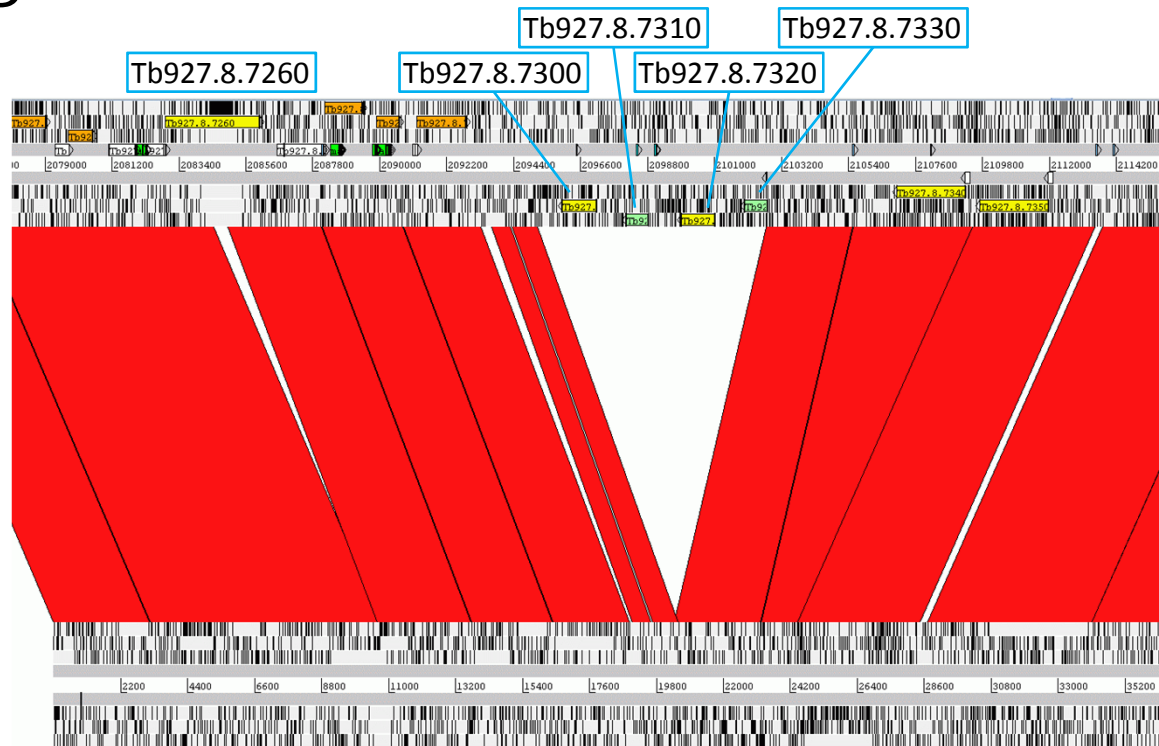

*T. evansi* STIB805 de novo contig

Supplement: S9 Fig — Absence of Tb927.8.7300 -.7330 in T. evansi STIB805. A: Mapping of T. evansi STIB805 reads against the Tb927.8.7300 -.7330 region in Tb927. Reads that could be uniquely mapped to the reference are colored blue, red or green. Reads that could be mapped to more than one position in the Tb927 reference were placed randomly and are colored yellow. B: Mapping of T. b. brucei TREU 927/4 Illumina reads to the Tb927 reference shown for comparison. C: Differential RPKM plot. RPKM values for T. b. brucei TREU 927/4 and T. evansi STIB805 Illumina reads mapped to the Tb927 reference were normalized for average coverage and the log2 ratio Tb/Te determined. X-axis: distance from the left end of the chromosome in kbp. The central line indicates the mean of the off-set (median of the ratios). The plot also reflects the internal deletion in gene Tb927.8.7260 (see D). D: Alignment of a 40.4 kb T. evansi STIB805 de novo contig to the Tb927.8.7300 -.7330 region on chromosome 8 of Tb927. Note also the deletion in the mid region of Tb927.8.7260 (upstream of Tb927.8.7300) that results in a frame-shift and predicted truncation of the encoded protein. Interestingly, this gene is annotated as “kinetoplast-associated” in TriTrypDB. Chromosome 8 in Tb927 contains a tandem repeat of two VSG-related (Tb927.8.7300/Tb927.8.7320) and two hypothetical genes (Tb927.8.7310/Tb927.8.7330). The former have been reported to be important for growth of bloodstream form T. b. brucei Lister 427 [89]. All four genes are absent in STIB805 and, according to TriTrypDB, from T. b. gambiense DAL972 as well. (PDF) [file pntd.0003404.s009.pdf]

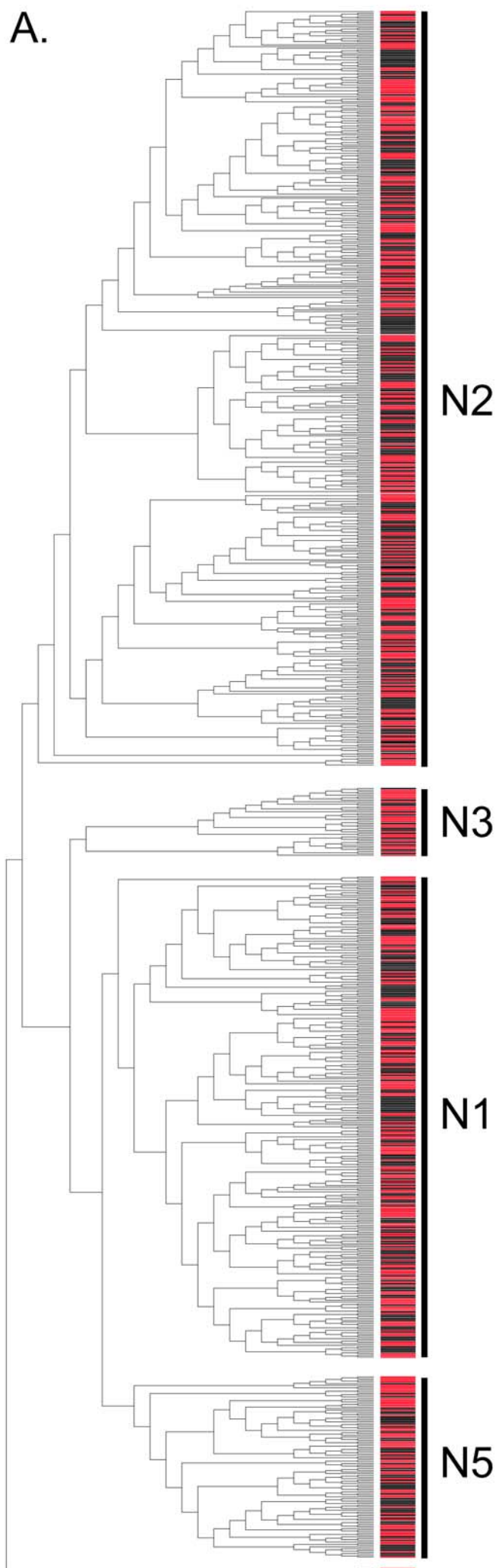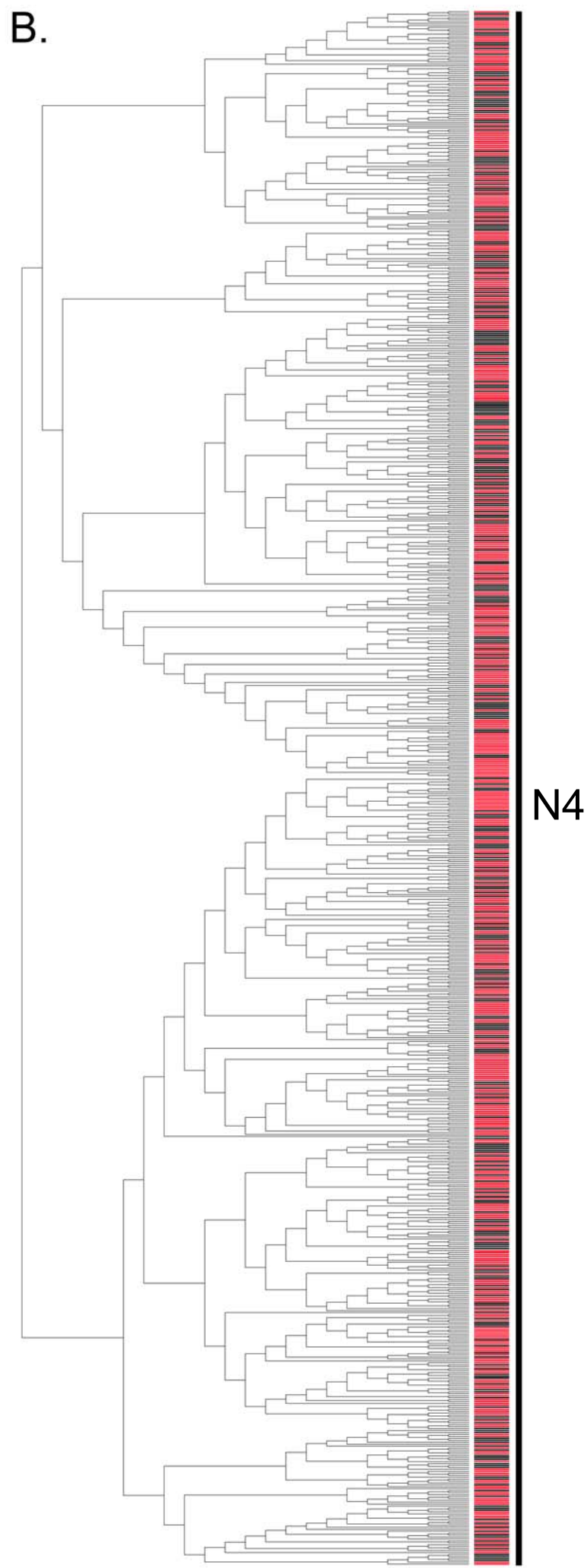

Supplement: S10 Fig — Neighbour-joining molecular cladograms of a-type and b-type VSG, with terminal nodes labeled as T. b. brucei TREU 927/4 (black) and T. evansi STIB805 (red). A. Cladogram showing the four recognized subgroups of a-type VSG (N1-3 and 5), estimated from a multiple protein sequence alignment of 470 characters using JTT matrix. B. Cladogram showing b-type VSG (N4) estimated from a multiple protein sequence alignment of 492 characters using JTT rate matrix. Both trees highlight the fact that T. evansi sequences are distributed throughout the T. brucei VSG tree without strain-specific clades. (PDF) [file pntd.0003404.s010.pdf]
