# Supplementary material for: Genome and Phylogenetic Analyses of Trypanosoma evansi Reveal Extensive Similarity to T. brucei and Multiple Independent Origins for Dyskinetoplasty
Source: PLoS Negl Trop Dis. 2015 Jan 8;9(1):e3404. doi: 10.1371/journal.pntd.0003404 (PMC4288722; doi:10.1371/journal.pntd.0003404)
Supplement: S11 Fig — Identification of a RoTat1.2 ortholog in T. evansi STIB805. A: Sequence alignment of bp 1-850 of an ORF on T. evansi STIB805 de novo contig 5566 with the T. evansi RoTat 1.2 gene (NCBI entry AF317914). B: Alignment of bp 851-1431 and 851-1412, respectively, of the same sequences. C: Alignment of bp 1223–1431 of contig 5566 ORF with bp 1138–1355 of the degenerate VSG Tb927.8.240. (PDF) [file pntd.0003404.s011.pdf]

A

```
ORF_contig5566        1 ATGCAAACCAAGGCGCTCGTTGGCGTACTCTTATTTGTACTGTATCGGAG    50
                        ||||||||||||||||||||||||||||||||||||||||||||||||||
RoTat1.2(AF317914)    1 atgcaaaccaaggcgctcgttggcgtactcttatttgtactgtatcggag    50

ORF_contig5566       51 CACAACGGATGCCGCCAATGTAGCTCTTAAAGGCAACGTCTGGAAGCCAT   100
                        ||||||||||||||||||||||||||||||||||||||||||||||||||
RoTat1.2(AF317914)   51 cacaacggatgccgccaatgtagctcttaaaggcaacgtctggaagccat   100

ORF_contig5566      101 TGTGCGAACTCGCGGCAGCGACCAGGAACGGGCCAAGCCACGGCACGGCG   150
                        ||||||||||||||||||||||||||||||||||||||||||||||||||
RoTat1.2(AF317914)  101 tgtgcgaactcgcggcagcgaccaggaacgggccaagccacggcacggcg   150

ORF_contig5566      151 CACTTCGCAGCGATCGAAAATAGCGTCGAAACGTACACTAAGTTAAAACT   200
                        ||||||||||||||||||||||||||||||||||||||||||||||||||
RoTat1.2(AF317914)  151 cacttcgcagcgatcgaaaatagcgtcgaaacgtacactaagttaaaact   200

ORF_contig5566      201 AAAGCTCTTGATTTACGCGGCGGCCAAAGGCAGCACCACCGAAGCAAGCG   250
                        ||||||||||||||||||||||||||||||||||||||||||||||||||
RoTat1.2(AF317914)  201 aaagctcttgatttacgcggcggccaaaggcagcaccaccgaagcaagcg   250

ORF_contig5566      251 CAGCAAGAGGGTTAGCAGCGGCCGCAGATAGACACATACGAGCAGCGGCC   300
                        ||||||||||||||||||||||||||||||||||||||||||||||||||
RoTat1.2(AF317914)  251 cagcaagagggttagcagcggccgcagatagacacatacgagcagcggcc   300

ORF_contig5566      301 ACCACGGCGAAAGACAAAAGCAGGGTAATTCTGCCCGCAGTTGCCTATGG   350
                        ||||||||||||||||||||||||||||||||||||||||||||||||||
RoTat1.2(AF317914)  301 accacggcgaaagacaaaagcagggtaattctgcccgcagttgcctatgg   350

ORF_contig5566      351 CGGCGAAGTCGCAGGGGCGATTTCATCGGCGCTAAAATTTCTAAAGCACG   400
                        ||||||||||||||||||||||||||||||||||||||||||||||||||
RoTat1.2(AF317914)  351 cggcgaagtcgcaggggcgatttcatcggcgctaaaatttctaaagcacg   400

ORF_contig5566      401 CGGTTGGCAACAGCAAGTTTTGTGTGGGCAAAGCCGACGGCACAAATGCC   450
                        ||||||||||||||||||||||||||||||||||||||||||||||||||
RoTat1.2(AF317914)  401 cggttggcaacagcaagttttgtgtgggcaaagccgacggcacaaatgcc   450

ORF_contig5566      451 GACGGTAACAACGAAATCGACGCGCTAGGGTGCGGCGAAGCCAACTATGA   500
                        ||||||||||||||||||||||||||||||||||||||||||||||||||
RoTat1.2(AF317914)  451 gacggtaacaacgaaatcgacgcgctagggtgcggcgaagccaactatga   500

ORF_contig5566      501 CACCTCGGCCCCAGGAGACAGCTACCTAGAGGGCGACATAAGCGCCGATG   550
                        ||||||||||||||||||||||||||||||||||||||||||||||||||
RoTat1.2(AF317914)  501 cacctcggccccaggagacagctacctagagggcgacataagcgccgatg   550

ORF_contig5566      551 GCTTCACAAAACTAACAGCCGTTGCAGCGGGCAATGGACATGTAGGAAGC   600
                        ||||||||||||||||||||||||||||||||||||||||||||||||||
RoTat1.2(AF317914)  551 gcttcacaaaactaacagccgttgcagcgggcaatggacatgtaggaagc   600

ORF_contig5566      601 AACACCTGCGGGGTGTTTAAAGCAATAACCGGCAACGACGGCGAGGCCGG   650
                        ||||||||||||||||||||||||||||||||||||||||||||||||||
RoTat1.2(AF317914)  601 aacacctgcggggtgtttaaagcaataaccggcaacgacggcgaggccgg   650

ORF_contig5566      651 CGGGATCAAAATCGCGACCAGCAACATCAAGGTGCACCTCGCACACGGCC   700
                        ||||||||||||||||||||||||||||||||||||||||||||||||||
RoTat1.2(AF317914)  651 cgggatcaaaatcgcgaccagcaacatcaaggtgcacctcgcacacggcc   700

ORF_contig5566      701 TAATCGAAGGCAAAGTTGACGACCAGCCAGAACGAGCAGAATTTTCCAAT   750
                        ||||||||||||||||||||||||||||||||||||||||||||||||||
RoTat1.2(AF317914)  701 taatcgaaggcaaagttgacgaccagccagaacgagcagaattttccaat   750

ORF_contig5566      751 AATTTCGGACAAGGAAAAGCACACCACACTGATTATTTAGGCCGAACACA   800
                        ||||||||||||||||||||||||||||||||||||||||||||||||||
RoTat1.2(AF317914)  751 aatttcggacaaggaaaagcacaccacactgattatttaggccgaacaca   800

ORF_contig5566      801 CGCAGCACTAATCAATCTGAAGAGGTTGGAAATGGAGAAGGTACCGGAAC   850
                        ||||||||||||||||||||||||||||||||||||||||||||||||||
RoTat1.2(AF317914)  801 cgcagcactaatcaatctgaagaggttggaaatggagaaggtaccggaac   850
```

B

```
ORF_contig5566      851 TCACAGAAGAAACCCTTAAGACTTTAGCAGACGACCCGGCCGCAACGGCA    900
                        ||||||||||||||||||||||||||||||||||||||||||||||||||
RoTat1.2(AF317914)  851 tcacagaagaaacccttaagactttagcagacgacccggccgcaacggca    900

ORF_contig5566      901 ACCCTAAACGTTGAGGAATGCGCACGAACAAGCAACAAGAAGATAACAAC    950
                        ||||||||||||||||||||||||||||||||||||||||||||||||||
RoTat1.2(AF317914)  901 accctaaacgttgaggaatgcgcacgaacaagcaacaagaagataacaac    950

ORF_contig5566      951 AACAGAACCACCGAAACCGCCCATAACCGAAAAATATTTTGGCAAGGACA   1000
                        ||||||||||||||||||||||||||||||||||||||||||||||||||
RoTat1.2(AF317914)  951 aacagaaccaccgaaaccgcccataaccgaaaaatattttggcaaggaca   1000

ORF_contig5566     1001 AGTCTAAAATCAAGGAGTTGTGGAACAATTTAAAAAAAGAGGAGATAGAA   1050
                        ||||||||||||||||||||||||||||||||||||||||||||||||||
RoTat1.2(AF317914) 1001 agtctaaaatcaaggagttgtggaacaatttaaaaaaagaggagatagaa   1050

ORF_contig5566     1051 GGAACAGAAGATGACACAACAAAGAAAGTAGCGCTAGAAACCGTCAACTC   1100
                        ||||||||||||||||||||||||||||||||||||||||||||||||||
RoTat1.2(AF317914) 1051 ggaacagaagatgacacaacaaagaaagtagcgctagaaaccgtcaactc   1100

ORF_contig5566     1101 GATCGACAAGCTGCAACAGGCATTGGAGTTTTACACAGCGCGAGCCGCTT   1150
                        ||||||||||.|||||||||||||||||||||||||||||||||||||||
RoTat1.2(AF317914) 1101 gatcgacaagttgcaacaggcattggagttttacacagcgcgagccgctt   1150

ORF_contig5566     1151 ACACAATAGAAAAGTTAAAAAAAGAAGTAGATAAGTTGCAAGCAGAATCA   1200
                        ||||||||||||||||||||||||||||||||||||||||||||||||||
RoTat1.2(AF317914) 1151 acacaatagaaaagttaaaaaaagaagtagataagttgcaagcagaatca   1200

ORF_contig5566     1201 GATGCAAAAAACAAAGCAAGCACAAAAGTTACTGAAACAGACGAAACTTG   1250
                        ||||||||||||||||||||||||||||||||||||||||.|||||||||
RoTat1.2(AF317914) 1201 gatgcaaaaaacaaagcaagcacaaaagttactgaaacagatgaaacttg   1250

ORF_contig5566     1251 CCAAAAGAAAGGAACAGGAGATAACTGCAAACCTCCATGCAAAGTTGTTG   1300
                        |.||||.|||||||||||||            ||..||||||||.||.|.
RoTat1.2(AF317914) 1251 cgaaaaaaaaggaacaggag------------ctgaatgcaaagatggtt   1288

ORF_contig5566     1301 GGAAA------------GGTGACACTACAAAGTGCAAATTGGAT------   1332
                        |.|||            |..||||.||.|||||||..|.|||||
RoTat1.2(AF317914) 1289 gtaaattgaccggcgttgtcgacaataaaaagtgcgtagtggatccggat   1338

ORF_contig5566     1333 ------AAGAAAGAAGAAAAAAACAGGCAGAAAACCAAGCAAGAGAGAAG   1376
                              ||.||.||||     .|.|||.||..|   ||||   .||.||.
RoTat1.2(AF317914) 1339 tttgtcaaaaaggaag-----tagagggagtta---aagc---ggaaaat   1377

ORF_contig5566     1377 GATAGGAAACCTGACTGCAGTAAATTTACCAACCACCAA-GCCTGTGAGG   1425
                        ||| |||||                  |.|||||||.|| .||  ..|||
RoTat1.2(AF317914) 1378 gat-ggaaa------------------aacaaccacaaacacc--acagg   1406

ORF_contig5566     1426 ATGTAA   1431
                        |.|.||
RoTat1.2(AF317914) 1407 aagcaa   1412
```

C

```
ORF_contig5566  1223 CAAAAGT---------TACTGAAACAGACGAAACTTGCCAAAAGAAAGGA   1263
                     ||.||.|         .||.||||||||||||||||||.|||| |||||.
Tb927.8.240     1138 CAGAACTCTCAACAAGAACAGAAACAGACGAAACTTGCGAAAA-AAAGGC   1186

ORF_contig5566  1264 ACAGGAGATAACTGCAAACCTCCATGCAAAGTTGTTGGGAAAGGTGACAC   1313
                     |||||||||||||||||||||||||||||||||||||||.||||||||.|
Tb927.8.240     1187 ACAGGAGATAACTGCAAACCTCCATGCAAAGTTGTTGGGGAAGGTGACGC   1236

ORF_contig5566  1314 TACAAAGTGCAAATTGGATAAGAAAGAAG-AAAAAAACAGGCAGAAAACC   1362
                     ..||||||||||||||.|||||.|||||| |||||||||||.||||||||
Tb927.8.240     1237 CGCAAAGTGCAAATTGTATAAGGAAGAAGTAAAAAAACAGGGAGAAAACC   1286

ORF_contig5566  1363 AAGCAAGAGAGAAGGATAGGAAACCTGACTGCAGTAAATTTACCAACCAC   1412
                     |||||||||||||.||||||||||||||||||||||||||||||||||||
Tb927.8.240     1287 AAGCAAGAGAGAAAGATAGGAAACCTGACTGCAGTAAATTTACCAACCAC   1336

ORF_contig5566  1413 CAAGCCTGTGAGGATGTAA   1431
                     .||||||.|||||||||||
Tb927.8.240     1337 TAAGCCTTTGAGGATGTAA   1355
```
