# Supplementary material for: Genome and Phylogenetic Analyses of Trypanosoma evansi Reveal Extensive Similarity to T. brucei and Multiple Independent Origins for Dyskinetoplasty
Source: PLoS Negl Trop Dis. 2015 Jan 8;9(1):e3404. doi: 10.1371/journal.pntd.0003404 (PMC4288722; doi:10.1371/journal.pntd.0003404)
Supplement: S5 Fig — Absence of IAO Tb927.9.16400 in T. evansi STIB805. A: Differential RPKM plot. RPKM values for T. b. brucei TREU 927/4 and T. evansi STIB805 Illumina reads mapped to the Tb927 reference were normalized for average coverage and the log2 ratio Tb/Te determined. X-axis: distance from the left end of the chromosome in kbp. The central line indicates the mean of the off-set (median of the ratios). The plot also indicates differences in the SL RNA and bloodstream alanine-rich protein (BARP) arrays between the reference and T. evansi STIB805. B: Alignment of a 6.2 kb T. evansi STIB805 de novo contig to the Tb927.9.14600 region on T. b. brucei TREU 927/4 chromosome 9. Note that Tb927.9.14600 (yellow box) is flanked by a direct repeat, as indicated by the overlap between the two regions of homology. No CDS corresponding to the IAO on T. b. brucei chromosome 9 (Tb927.9.14600) could be found in T. evansi STIB805, and a 6.2 kb de novo contig spanning that region supports the absence of that gene. Analysis of the T. b. gambiense DAL972 genome similarly found that Tb927.9.14600 is absent from its genomic repertoire, and it is also absent from numerous other T. brucei strains [33]. To our knowledge, the function of the IAO genes is presently unknown. (PDF) [file pntd.0003404.s005.pdf]

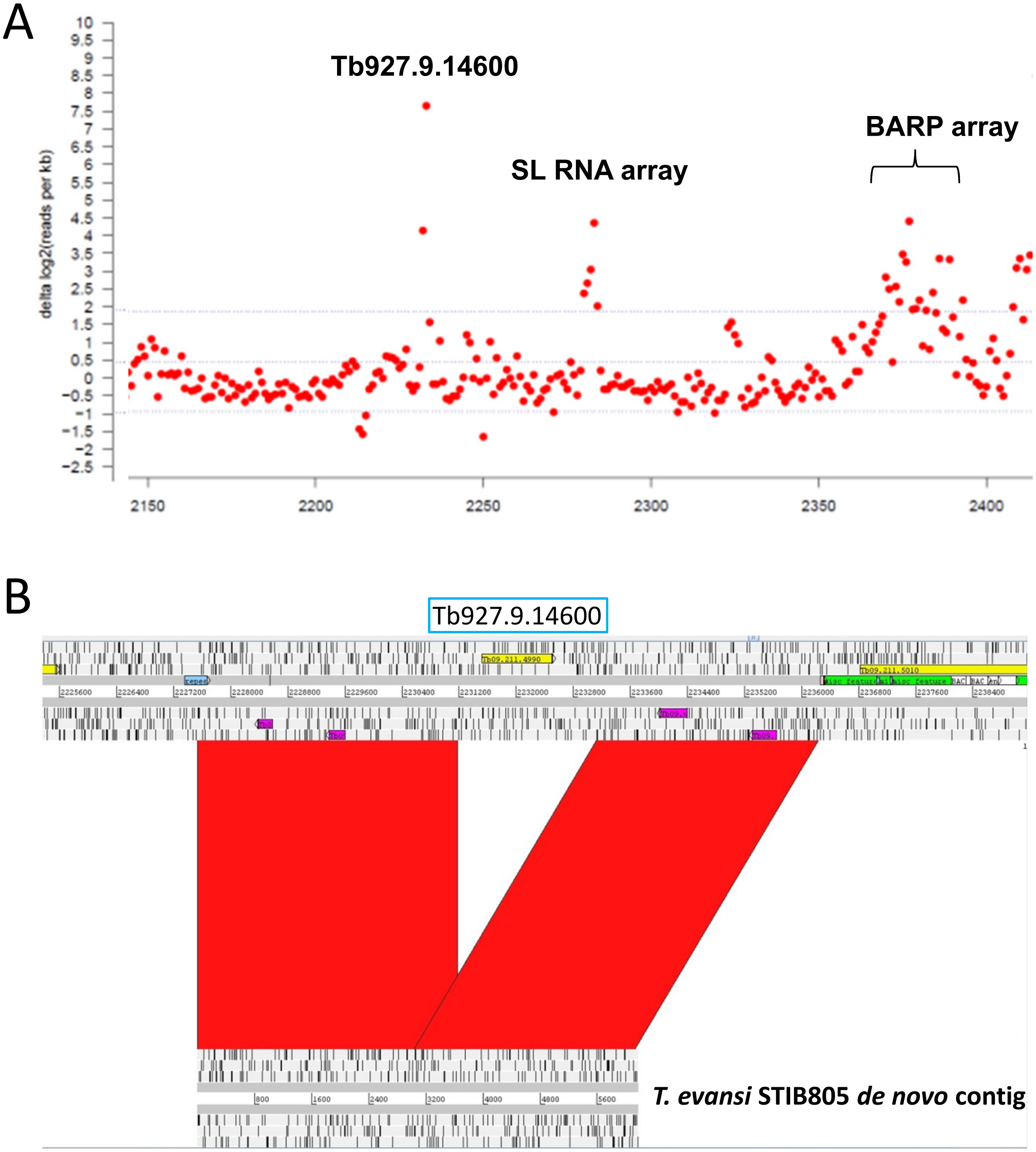
A
Tb927.9.14600
SL RNA array
BARP array
delta log2(reads per kb)
B
Tb927.9.14600
T. evansi STIB805 de novo contig
